# A Combinatorial Code for Splicing Silencing: UAGG and GGGG Motifs

**DOI:** 10.1371/journal.pbio.0030158

**Published:** 2005-04-19

**Authors:** Kyoungha Han, Gene Yeo, Ping An, Christopher B Burge, Paula J Grabowski

**Affiliations:** **1**Department of Biological Sciences, University of PittsburghPittsburgh, PennsylvaniaUnited States of America; **2**Department of Brain and Cognitive Sciences, Massachusetts Institute of TechnologyBoston, MassachusettsUnited States of America; **3**Department of Biology, Massachusetts Institute of TechnologyBoston, MassachusettsUnited States of America; University of Massachusetts Medical SchoolUnited States of America

## Abstract

Alternative pre-mRNA splicing is widely used to regulate gene expression by tuning the levels of tissue-specific mRNA isoforms. Few regulatory mechanisms are understood at the level of combinatorial control despite numerous sequences, distinct from splice sites, that have been shown to play roles in splicing enhancement or silencing. Here we use molecular approaches to identify a ternary combination of exonic UAGG and 5′-splice-site-proximal GGGG motifs that functions cooperatively to silence the brain-region-specific CI cassette exon (exon 19) of the glutamate NMDA R1 receptor *(GRIN1)* transcript. Disruption of three components of the motif pattern converted the CI cassette into a constitutive exon, while predominant skipping was conferred when the same components were introduced, de novo, into a heterologous constitutive exon. Predominant exon silencing was directed by the motif pattern in the presence of six competing exonic splicing enhancers, and this effect was retained after systematically repositioning the two exonic UAGGs within the CI cassette. In this system, hnRNP A1 was shown to mediate silencing while hnRNP H antagonized silencing. Genome-wide computational analysis combined with RT-PCR testing showed that a class of skipped human and mouse exons can be identified by searches that preserve the sequence and spatial configuration of the UAGG and GGGG motifs. This analysis suggests that the multi-component silencing code may play an important role in the tissue-specific regulation of the CI cassette exon, and that it may serve more generally as a molecular language to allow for intricate adjustments and the coordination of splicing patterns from different genes.

## Abstract 2

Many genes are alternatively spliced, but the signals that regulate the process are unclear. These authors have found a sequence motif that appears to function at many alternatively spliced genes

## Introduction

Alternative pre-mRNA splicing is a major determinant of the protein functional diversity underlying human physiology, development, and behavior [1]. This process combines exonic sequences in various arrangements to generate two or more mRNA transcripts from a single gene. Splicing patterns are inherently flexible, with variations observed in different cells and tissues and at different stages of development [2]. Inducible changes in splicing pattern can also occur as a function of cell excitation in neuronal systems, T cell activation, heat shock, or cell cycle changes [3,4,5,6]. Thus, a central problem is to understand the combinatorial mechanisms that adjust splicing patterns in different biological systems. A related issue is to understand how splicing errors, including alterations in splicing patterns, arise from inherited mutations or polymorphisms and contribute to human disease [7,8,9].

Splicing decisions occur in the context of the spliceosome, a highly complex molecular machine containing the small nuclear ribonucleoprotein particles U1, U2, and U4/U5/U6, and a host of protein factors [10,11,12]. Spliceosome assembly occurs in a stepwise fashion to recognize the appropriate splice sites, to fashion the small-nuclear-ribonucleoprotein-particle-based catalytic activity, and to couple the splicing process with transcription, 3′ end formation, and nuclear export. Exon definition, or recognition of the exon as a unit, occurs early in spliceosome assembly, and its efficiency depends upon the strengths of the adjacent splice sites, as well as auxiliary splicing regulatory elements.

RNA control elements, which are distinct from the canonical splice sites, include the positive-acting exonic splicing enhancers (ESEs) and intronic splicing enhancers, and the negative-acting exonic splicing silencers (ESSs) and intronic splicing silencers [8,13,14,15,16,17]. In order to achieve 100% inclusion of the exon in the processed mRNA, constitutive exons generally require some combination of ESEs in addition to the adjacent splice sites. Serine-arginine-rich (SR) protein factors are important mediators of splicing enhancement in both constitutive and alternative splicing. These proteins recognize ESE motifs through their RNA binding domains, and recruit splicing factors or interact with splice sites via interactions with their RS domains [18,19,20].

Alternative splicing affects the majority of human protein coding genes [21,22], but the molecular control mechanisms are poorly understood. Molecular dissection of a handful of prototypical alternatively spliced genes has shown that cassette exons are included at a frequency that depends on their complex arrangement of positive and negative RNA control elements. It is thought that combinatorial control, which involves the integrated actions of multiple RNA control elements and protein regulatory factors, is the basis of tissue-specific patterns of splicing. Many protein factors of the SR protein and heteronuclear ribonucleoprotein (hnRNP) protein families have been implicated in these mechanisms, and some of their expression patterns are tissue-specific. The polypyrimidine tract binding protein (PTB/hnRNP I), for example, plays important roles in mechanisms of negative control important for brain- and muscle-specific splicing events. Current evidence indicates that PTB/hnRNP I takes part in silencing by recognizing RNA elements containing UCUU and related motifs, and, through protein oligomerization, blocks recognition of the exon by the normal splicing machinery [23]. The hnRNP A1 protein has also been implicated in a variety of cellular and viral splicing silencing mechanisms through its cooperative recognition of U AGGG[U/A] and related motifs [24].

The CI cassette exon (exon 19) of the *GRIN1* transcript (NMDA-type glutamate receptor, NR1 subunit) is a valuable model to study mechanisms of regulation because of its striking patterns of tissue-specific splicing and developmental regulation in the rat brain [25,26]. (Note that the CI exon is referred to as E21 in these previous studies.) The CI exon is prominently included in the forebrain, and prominently skipped in the hindbrain, but the control mechanisms underlying these patterns are poorly understood. The RNA binding protein NAPOR/CUGBP2 is thought to positively regulate this exon since this factor promotes CI cassette exon inclusion in co-expression assays, and because its tissue-specific expression correlates with the spatial distribution of mRNA transcripts containing the CI exon in rat brain [26]. In mammals, NMDA-type glutamate receptors are assembled from *GRIN1* (NR1) and *GRIN2A* (NR2) subunits, and they play highly important roles impacting learning and memory functions in the brain. Alternative splicing is used extensively for the generation of the brain-specific *GRIN1* transcripts, and CI exon inclusion affects the trafficking of NMDA receptors to the synapse [27,28].

In many cases tissue-specific exon inclusion is modulated by combinations of sequence motifs acting cooperatively or antagonistically [29]. An understanding of the essential ingredients for splicing silencing should allow de novo identification of skipped exons from genomic sequence. Here molecular approaches were used to identify sequences responsible for silencing the CI cassette exon, and this analysis was extended using computational methods to explore the distribution and functional relevance of the identified motifs in mammalian genomes. It is a paradox that the CI cassette exon undergoes predominant exon skipping in particular regions of the brain, since its adjacent splice sites match well to consensus patterns. In our previous study, the downstream intron was shown to play a role in silencing, but the factors involved were not defined [26].

Here we define a ternary sequence code—two exonic UAGGs and a 5′-splice-site-proximal GGGG—that imposes silencing on an inherently strong CI cassette exon. We further extend this analysis to investigate the roles of hnRNP proteins and the generality of this type of mechanism genome-wide using molecular and bioinformatics approaches. The association of exon silencing with a UAGG and GGGG motif pattern in human and mouse exons otherwise unrelated to the CI cassette supports the generality of this mechanism, and this is consistent with the demonstrated flexibility in the spatial positioning of the UAGG components of the code.

## Results

### A 5′-Splice-Site-Proximal GGGG and Two Exonic UAGG Motifs Are Required in Combination for Silencing of a Brain-Region-Specific Exon

The 5′ splice site of the CI cassette exon is atypical because of an adjacent GGGG motif, which is conserved in human, rat, and mouse *GRIN1* genes. GGGG motifs in the first ten nucleotides of human introns are generally infrequent (see below). In the case of the CI cassette exon, the GGGG motif is immediately adjacent to the U1 small nuclear RNA complementary region of the 5′ splice site, and the overall complementarity of the 5′ splice site (6 bp) is typical for mammals (6 to 7 bp), including all of the most highly conserved positions (−1 to +5).

The role of the GGGG motif in splicing silencing of the CI cassette exon was examined by generating site-directed mutations in nucleotides +6, +7, and +8 of the intron. These mutations were designed so as not to disrupt the U1 small nuclear RNA complementary nucleotides, which include the last nucleotide of the CI exon and the first five nucleotides of the adjacent intron. Splicing assays involved transfecting splicing reporters into non-neuronal mouse myoblasts (C2C12 cells), followed by measurement of the levels of the exon-included and exon-skipped products by RT-PCR relative to the wild-type sequence.

Each mutation in the GGGG motif led to a dramatic increase in exon inclusion (Figure 1A). The strongest effects were observed when the GGG at +6 to +8 was converted to CCC (mutation 5m2) or AUA (5m4), which resulted in an approximately 4-fold increase in exon inclusion, compared to the wild-type sequence. Even a point mutation (5m9) resulted in a 3-fold increase in exon inclusion. Thus, the GGGG motif plays an important role in the silencing mechanism. Additional sequence changes upstream and downstream of the GGGG motif had only modest effects on splicing. For example, mutations 5m1, 5m13, and 5m14 were designed to test potential RNA secondary structures involving the GGGG motif and complementary intron sequences. The modest changes in the splicing pattern resulting from these mutations do not support a significant role in splicing for these hypothetical structures.

**Figure 1:**
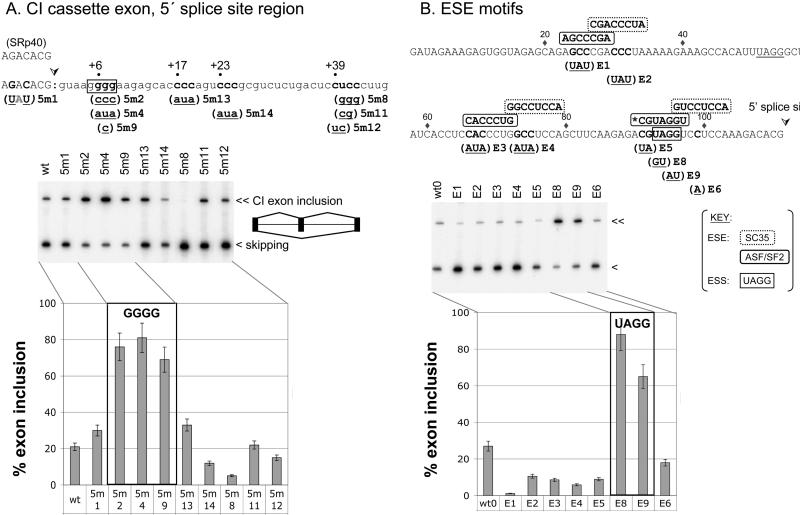
Exonic UAGG and 5′ Splice Site GGGG Motifs Are Required in Combination for Silencing of the CI Cassette Exon (A) A GGGG splicing silencer motif at the 5′ splice site. Top: Sequence of the 5′ splice site region (5′ to 3′) with exonic (uppercase) and intronic (lowercase) nucleotides. Numbering is relative to the first nucleotide of the intron. Arrowhead indicates 5′ splice site. A predicted SRp40 motif overlying the last seven bases of the exon is indicated. Engineered mutations and names of splicing reporters are indicated immediately below the affected nucleotides. Effect of mutations on the pattern of splicing is shown in a 5′ to 3′ arrangement (gel panel and graph). All splicing reporter plasmids have a three-exon structure in which CI is the middle exon (in the schematic, vertical bars indicate exons and horizontal lines indicate introns). Splicing reporter plasmids were expressed in vivo in mouse C2C12 cells, and splicing patterns assayed by radiolabeled RT-PCR of cellular RNA harvested from the cells. PCR primers are specific for the flanking exons. Results of multiple experiments are shown graphically as the average percent of exon included in product (y-axis) for each splicing reporter construct (x-axis). (B) Analysis of ESE motifs. An exonic UAGG splicing silencer motif overlaps an ASF/SF2 motif. Sequence of the CI exon (5′ to 3′) is shown, with engineered mutations (underscored) and names of splicing reporters indicated immediately below the affected nucleotides (bold). Numbering is relative to the first nucleotide of the exon. Predicted ESE motifs for ASF/SF2 and SC35 are highlighted above the exonic sequence as indicated in brackets. The UAGG motif required for silencing (boxed) is indicated below the overlapping ASF/SF2 motif (asterisk). Effect of mutations on the in vivo pattern of splicing is shown in a 5′ to 3′ arrangement (gel panel and graph). Error bars in (A) and (B) represent standard deviations.

Other than the GGGG motif at the 5′ splice site, the sequence of this intronic region is devoid of guanosine-rich sequences. Strikingly, introduction of a GGG at intron positions +40 to +42 (5m8) resulted in a 5-fold decrease in exon inclusion. In contrast, two overlapping mutations that did not generate guanosine-rich motifs had little or no effect on the splicing pattern (5m11 and 5m12). Thus, in this context the introduction of a second intronic GGG cluster can shift the splicing pattern toward nearly complete exon skipping.

The possibility that sequences within the CI cassette exon itself might contribute to the silencing mechanism was also explored. Either a scarcity of ESE sequences within the CI cassette exon might weaken exon definition, or the presence of exonic ESS sequences might enforce silencing. A model for the arrangement of ESE motifs in the CI cassette exon was based on the high-affinity sequence-recognition sites for known SR family splicing factors (Figure 1B, top). Mutations were then made in the ASF/SF2 ( AGCCCGA, CACCCUG, and CGUAGGU) and SC35 ( CGACCCUA, GGCCUCCA, and GUCCUCCA) motifs to test predictions of this model, anticipating that reduced exon inclusion should result from the disruption of functional ESE motifs.

The results of these experiments show that most of the mutations decreased exon inclusion, consistent with ESE function (mutations E1, E2, E3, E4, E5, and E6; Figure 1B). In contrast, a pair of double point mutations in a UAGG sequence beginning at position 93 of the exon generated a substantial increase in exon inclusion, indicative of a silencing role for this sequence (E8 and E9; Figure 1B). Note that the overlapping ASF/SF2 motif is disrupted by the E9 mutation, but the E8 mutation generates a different ASF/SF2 motif. An additional six-nucleotide mutation (CAUCGU) that eliminates the ASF/SF2 motif at this position also resulted in a strong increase in exon inclusion (K. H. and P. J. G., unpublished data). These results show that the position 93 UAGG motif functions in C2C12 cells primarily as a silencer rather than as part of an ASF/SF2 motif. These results suggested the possible involvement of the splicing repressor hnRNP A1 based on the similarity of the UAGG motif to the hnRNP A1 high-affinity binding sequence U AGGG[A/U] determined previously by SELEX experiments [30].

### A Motif Pattern for Strong Splicing Silencing: Analysis of Copy Number and Position Effects in Neuronal and Non-Neuronal Cells

The presence of two natural UAGG motifs in the CI cassette exon raised the question of how silencing might be affected by changes in the number of exonic UAGGs. The number and position of UAGG motifs in the CI cassette exon were altered in the context of the wild-type splicing reporter (wt0) and the effects tested in neuronal (PC12) and non-neuronal (C2C12) cell lines (Figure 2). One set of mutations varied the position of the 5′-splice-site-proximal UAGG by disrupting the original motif at position 93 of the exon, and by introducing a new UAGG motif at positions 11, 76, and 100 (splicing reporters E10, E11, and E20). These position variations had small effects on the pattern of splicing, with exon skipping predominating in both cell lines (Figure 2, lanes 1–4 and 15–18). The effect of a single UAGG was then examined at different positions of the exon (splicing reporters E8, E13, E14, E15, and E21). The resulting splicing patterns uniformly showed an increase in exon inclusion, and these effects were essentially independent of position (Figure 2, lanes 5–9 and 19–23). It was also evident that the level of exon inclusion was higher in C2C12 than in PC12 cells, suggesting that there may be differences in splicing factors that mediate or antagonize silencing in the two cell lines. Nonetheless, each cell line exhibited a similar trend—stronger exon silencing associated with increased copy number of exonic UAGGs. Thus, splicing silencing of the CI cassette exon depends critically on the number of UAGG motifs in the exon, but less so on their relative positions. To further test the prediction that the strength of splicing silencing is linked to the number of UAGGs in the exon, a third UAGG was introduced at position 11 of the exon (splicing reporter E18). As a result, the level of exon inclusion decreased to approximately 0% in both cell lines in agreement with this prediction (lanes 11 and 25).

**Figure 2:**
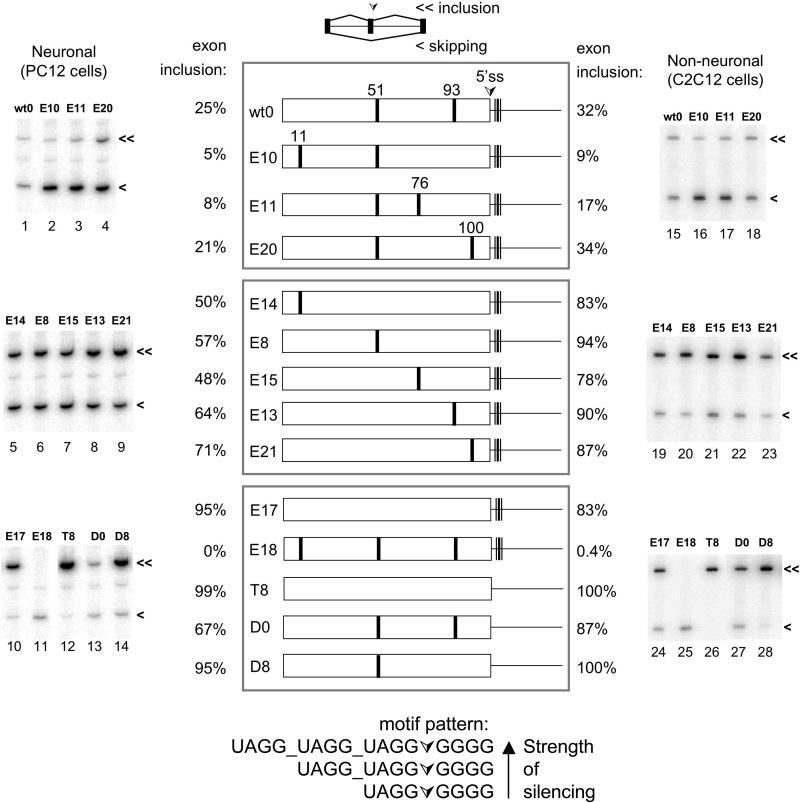
Effect of Number and Position of CI Cassette Exon Splicing Silencer Motifs Splicing reporters were constructed with variations in the number and position of UAGG and/or GGGG motifs. Three sets of schematics (boxed at center) illustrate the CI cassette exon and adjacent 5′ splice site region with positions of exonic UAGG (black vertical bars) and 5′ splice site GGGG (grey vertical stripe) motifs. Splicing reporter names are indicated at left. Vertical arrowhead indicates 5′ splice site. Each splicing reporter was generated by site-directed mutagenesis from parent plasmid wt0. Natural UAGG positions 51 and 93 represent the starting position of the motif relative to the first base of the exon. Engineered UAGG positions 11, 76, and 100 are also indicated (see schematic in center box at top). Sequence changes of the mutations are underscored: 11, GUGG→UAGG; 51, UAGG→AUGG; 76, CCAG→UAGG; 93, UAGG→GUGG; 100, UCCAA→UAGGC. Representative splicing patterns in PC12 cells (left gel panels) and C2C12 cells (right gel panels) are shown together with average percent exon inclusion values. The correlation between motif pattern and strength of splicing silencing is summarized (bottom). Exon-included (double arrowheads) and exon-skipped (single arrowheads) products are indicated.

The role of the 5′-splice-site-proximal GGGG motif was examined independently by generating exons lacking the two natural UAGG motifs in the presence and absence of the GGGG motif (splicing reporters E17 and T8, respectively; Figure 2, lanes 10, 12, 24, 26). The GGGG motif had a small silencing effect in both cell lines in the absence of the exonic UAGGs (compare E17 and T8; lanes 10 versus 12, and 24 versus 26). By contrast, silencing was reduced substantially when the GGGG motif was disrupted by mutation in the presence of intact UAGGs: exon inclusion increased significantly in PC12 cells (from 25% to 67%; compare wt0 and D0: Figure 2, lanes 13 and 14), and a similar trend was observed in C2C12 cells (from 32% to 87%; Figure 2, lanes 27 and 28). Note that mutant D0 contains two intact UAGGs, but lacks the GGGG motif. Thus, the GGGG motif acts cooperatively with the exonic UAGGs in both of these cell lines. Together these results show that, for the CI cassette, multiple exonic UAGGs combined with a 5′-splice-site-proximal GGGG function cooperatively to specify silencing of an otherwise strong exon.

### The 5′-Splice-Site-Proximal GGGG Motif Is Involved in Silencing by hnRNP A1 and Anti-Silencing by hnRNP H

Next we sought to identify protein factors that interact directly with the UAGG and GGGG motifs in order to guide empirical tests for their roles in splicing silencing. GTP-labeled RNA substrates were subjected to UV crosslinking in HeLa nuclear extracts under in vitro splicing conditions. These experiments showed pronounced crosslinking to a protein doublet in the vicinity of 50 kDa for RNA substrates containing the intact GGGG motif (cs1 and 3h1; Figure 3A, lanes 1 and 3). By contrast, a point mutation in the GGGG motif largely disrupts protein binding (cs3 and 3h3; Figure 3A, lanes 2 and 4). Because the apparent molecular weights of these proteins and the guanosine-rich binding specificity [31] suggested the involvement of hnRNP H/H′ and F proteins, relevant antibodies were obtained for immunoprecipitation experiments. These results identified the bottom band of the doublet as hnRNP F (Figure 3A, lanes 5–7), whereas the upper band corresponded to hnRNP H/H′ (Figure 3A, lanes 8 and 9). Although the hnRNP F antibody is highly specific, the H/H′ antibody crossreacts with hnRNP F, which is 95% identical to H/H′ at the protein sequence level. Control reactions (Figure 3A, lanes 10 and 11) show the background level precipitated with preimmune serum (lane 10).

**Figure 3:**
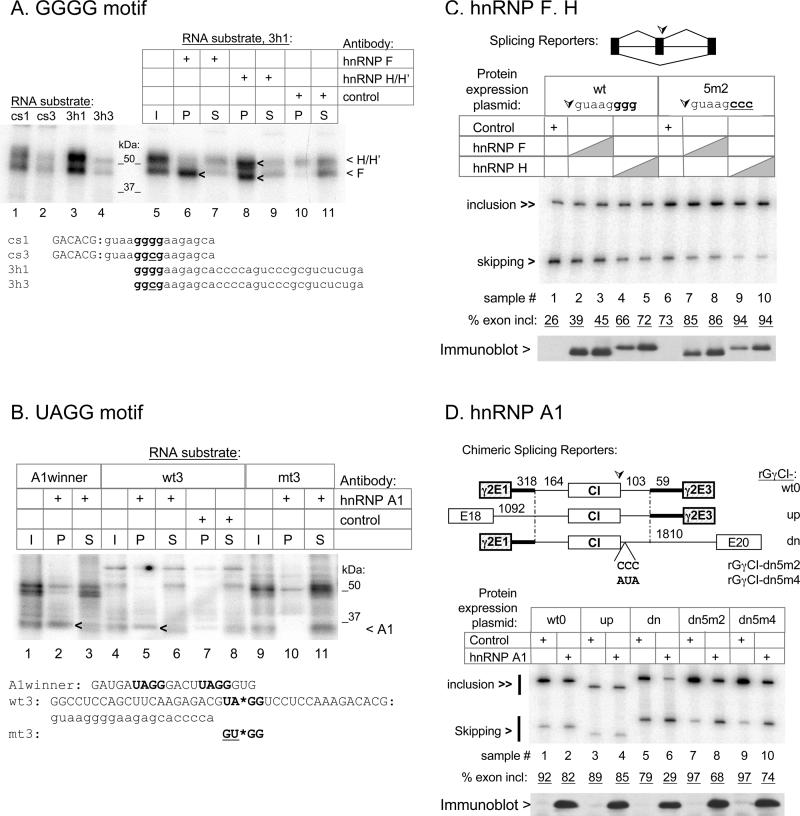
Identification and Functional Roles of Protein Factors That Bind to GGGG and UAGG Motifs (A) Detection of protein binding to the 5′ splice site GGGG motif by UV crosslinking in HeLa nuclear extract. Wild-type (cs1 and 3h1) and mutant (cs3 and 3h3) RNA substrates were internally labeled at guanosine nucleotides; mutations are underscored. Pattern of UV crosslinking is shown following RNase digestion and SDS-PAGE (lanes 1–4). Immunoprecipitation reactions (lanes 5–11) contained the 3h1 substrate together with antibody specific for hnRNP F or H/H′; control samples contained preimmune rabbit serum. Gel panel shows the pellet (P), supernatant (S), and input (I) of the immunoprecipitation reactions following SDS-PAGE. The positions of hnRNP H/H′ and F (arrowheads) and protein molecular weight standards (in kilodaltons) are indicated. The hnRNP F and H/H′ antibodies were a gift of C. Milcarek. (B) UV crosslinking of exonic position 93 UAGG motif in HeLa nuclear extract. RNA substrates were prepared with a single radiolabeled nucleotide as indicated by the asterisk; sequences are shown (bottom). The wild-type (wt3) and mutant (mt3) substrates are identical except for the underscored mutation. The A1winner substrate corresponds to the high-affinity hnRNP A1 binding sequence previously identified by SELEX. The position of hnRNP A1 is indicated (arrowhead). Monoclonal antibody 9H10 was a gift of G. Dreyfuss. (C) Exon inclusion is enhanced by co-expression of hnRNP F or H. Gel panel shows splicing pattern resulting from co-transfection of wild-type (wt) or mutant (5m2) splicing reporter with hnRNP F or H expression plasmid; splicing reporters are identical to those shown in Figure 1A. Control samples were transfected with empty vector; grey wedge indicates two levels (4 and 6 μg) of protein expression plasmid. Arrowhead indicates 5′ splice site. For immunoblot verification of transfected protein expression (bottom), nuclear extracts from transfected cells were separated by SDS-PAGE, transferred to nylon membrane, and developed with an antibody specific for the Xpress tag at the N-terminus of each pcDNA–protein sample. Raw percent exon inclusion values are shown below gel image. (D) Silencing effect of hnRNP A1 requires the intact 5′ splice site GGGG motif and full-length downstream intron. Structures of chimeric splicing reporters are shown in which the CI cassette exon and intron flanks were introduced into an unrelated splicing reporter containing sequences from the GABA_A_ receptor γ2 transcript: rGγCI-wt0 (both introns truncated), -up (full-length upstream intron, truncated downstream intron), and -dn (truncated upstream intron, full-length downstream intron). Numbers above indicate length of each intron segment in nucleotides. Arrowhead indicates 5′ splice site. The splicing reporters rGγCI-dn5m2 and -dn5m4 contain the full-length downstream intron with 5′ splice site mutations of Figure 1A. Gel panel shows splicing pattern resulting from co-transfection of splicing reporter with hnRNP A1 expression plasmid or vector control. Immunoblot verification of transfected protein expression (bottom) is as described in (C).

Proteins that interact directly with the exonic UAGG motif were identified similarly, except that the RNA substrates contained a single radioactive label in the middle of the UAGG. Even with a single radioactive label, multiple proteins were observed to crosslink to the wild-type substrate, wt3, under splicing conditions (Figure 3B, lane 4). To examine hnRNP A1 binding, the SELEX-derived consensus sequence, A1winner, was also tested in parallel. A low efficiency of UV crosslinking of hnRNP A1 has been observed previously [30]. The A1winner contains two U AGGGA sequences, and was found to crosslink to hnRNP H/H′ and F, in addition to A1 (Figure 3B, lane 1; data not shown). These results show that A1 is immunoprecipitated as an approximately 35-kDa protein from the wt3 sample, as was the case for the A1winner (Figure 3B, lanes 1–8). A control substrate, mt3, with a dinucleotide mutation in the UAGG showed little or no immunoprecipitation of crosslinked A1 (Figure 3B, lanes 9–11). Thus, these results confirm that hnRNP A1 binds directly to the UAGG motif in the context of the CI cassette exon sequence.

In order to investigate the functional roles of hnRNPs F, H, and A1 in the silencing mechanism, each protein was co-expressed with splicing reporters containing the CI cassette exon, and effects on the splicing pattern were monitored. For the wild-type splicing reporter containing an intact GGGG motif, overexpression of hnRNP F or H was found to enhance CI exon inclusion relative to the pcDNA control (Figure 3C, lanes 1–5). These effects were reduced but not eliminated in the presence of the 5m2 splicing reporter, which lacks the GGGG motif (Figure 3C, lanes 6–10). These results rule out a role in silencing of the CI exon for hnRNP F and H, and instead support an anti-silencing role for these factors.

Next we asked whether the silencing role of the GGGG motif is mediated through hnRNP A1, since the 5′ splice site of the CI cassette exon is related to the A1 consensus binding motif (ACG:GU AAGGGGAA [colon defines 5′ splice site] versus U AGGG[A/U]). These experiments also examined the effects of portions of the flanking introns, since our previous study demonstrated a role for the downstream intron in this silencing mechanism. Chimeric splicing reporters contained the CI cassette exon and various portions of the flanking introns inserted between exons 1 and 3 of the GABA_A_ receptor γ2 subunit (Figure 3D). When the complete downstream intron was present, co-expression of hnRNP A1 reduced exon inclusion from 78.8% to 29.1%, nearly a 3-fold effect (Figure 3D, lanes 5 and 6). In this context, the silencing effect of hnRNP A1 depends upon the intact downstream intron, since the silencing effect was substantially reduced when most of the downstream intron was removed (rGγCI-wt0 and rGγCI-up; Figure 3D, lanes 1–4). The role of the 5′ splice site GGGG motif was then examined in the context of the rGγCI-dn reporter by introducing mutations 5m2 and 5m4, which destroy the guanosine cluster. The ability of hnRNP A1 to induce splicing silencing was reduced significantly by these mutations, suggesting that A1 is involved in mediating the cooperative effects of the GGGG motif (rGγCI-dn5m2 and rGγCI-dn5m4 Figure 3D, lanes 7–10).

### Combinations of UAGG and GGGG Motifs Are Associated with cDNA- and EST-Confirmed Skipped Exons in the Human and Mouse Genomes

We next sought to determine the extent to which the CI cassette silencing motif pattern is associated with exon skipping (partial or complete) in the human and mouse genomes. For this analysis, over 90,000 human and mouse orthologous exon pairs were divided into two datasets based on the presence or absence of one or more UAGG motifs at any position in the exon (but not overlapping the splice sites) and a GGGG motif within bases 3–10 of the adjacent downstream intron (Figure 4). The percentage of alternatively spliced (skipped) exons in each of these datasets was then determined by use of large-scale, high-stringency alignments of available cDNAs and ESTs to the corresponding genomic loci (see Materials and Methods). If the motif pattern functions generally in splicing silencing, the frequency of exon skipping should be higher in the group of exons containing the UAGG and GGGG motif pattern, compared to those without.

**Figure 4:**
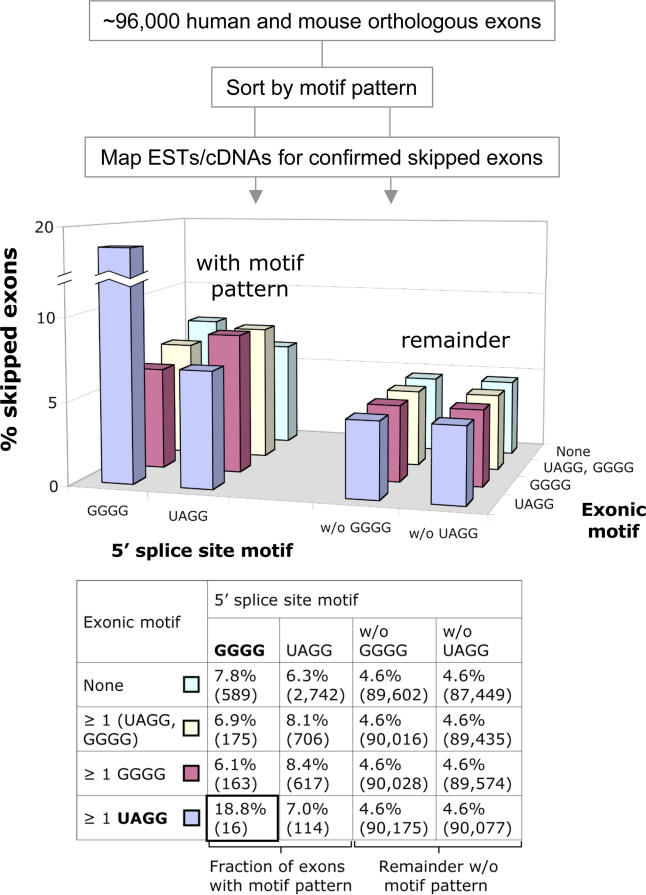
Computational Analysis of UAGG and GGGG Motif Patterns Reveals Association with Exon Skipping Genome-Wide At the top is a flow chart for the computational analysis used to illustrate the procedure used to identify human exons with and without the CI cassette silencer motif pattern (≥1 exonic UAGG and a 5′-splice-site-proximal GGGG), followed by the determination of the percentage of confirmed skipped exons in each group. The reciprocal pattern (≥1 exonic GGGG and a 5′ splice site UAGG) and related variants were analyzed for comparison as indicated in the graph and table. The graph (middle) shows exons with the motif pattern on the left and the remaining exons without the pattern (w/o) on the right; x-axis, 5′ splice site motif; y-axis, percent confirmed skipped exons; z-axis, exonic motif. Confirmed skipped exons were defined as those skipping events supported by 20 or more individual cDNA and/or EST entries. Exonic motifs were allowed at any position within the exon, but not overlapping the splice sites, and the 5′ splice site motif was restricted to bases 3–10 of the intron. Only exons of 250 nucleotides or fewer were considered. The table (bottom) shows, for each motif pattern, the percentage of confirmed skipped exons within that group (as shown in the graph) and the number of exons in the group (in parentheses). The CI cassette silencer motif pattern is boxed.

In these searches we considered exons of typical size (≤250 bases), and we required each component of the motif pattern to be conserved in sequence and position in the human and mouse orthologous exons. Using these stringent criteria, 16 exons (0.018%) contained the motif pattern, and of these, three were confirmed skipped exons (18.75%). The remaining 90,175 exons (99.98%) lacked the conserved motif pattern, and of these, 4,173 (4.63%) were confirmed skipped exons. The difference in the percentage of skipped exons in these two datasets was significant (*p* < 0.05). When exon length was not constrained, the fraction of skipped exons with the motifs was slightly lower (15.8%), but still significant (*p* < 0.05). When this analysis was repeated without requiring conservation of the motif pattern, 227 exons (0.24%) contained the motif pattern, and of these, 18 (7.9%) were confirmed skipped exons (*p* < 0.05). The remaining 96,292 exons (99.76%) lacked the motif pattern, and of these 4,441 (4.61%) were confirmed skipped exons.

Variations of the CI cassette motif pattern were also analyzed. The reciprocal pattern, one or more GGGG motifs in the exon and a UAGG motif in bases 3–10 of the intron, also showed enrichment for confirmed skipped exons (8.4%) compared to those without this pattern (4.6%) (*p* < 0.001). Moreover, the occurrence of a 5′ splice site GGGG by itself was found to be associated with exon skipping: exons containing the GGGG motif in bases 3–10 of the intron but lacking UAGG and GGGG within the exon showed a significantly higher rate of exon skipping (7.8%) compared to those without the GGGG intronic motif (4.6%) (*p* < 0.001). Moving the position of the GGGG motif slightly downstream to bases 11–20 of the intron reduced the fraction of skipped exons observed to background levels (4.6%). Taken together, these data suggest that the close proximity (or overlap) of the GGGG motif to the 5′ splice site may be generally important in silencing, perhaps by limiting binding of U1 or U6 small nuclear ribonucleoprotein particles.

### Underrepresentation of UAGG in Constitutive Exons, and Overrepresentation in Skipped Exons

Underrepresentation of UAGG in constitutively spliced exons and overrepresentation in skipped exons would be expected if this motif frequently plays a role in splicing silencing. To test this idea, approximately 5,000 known human cDNAs were downloaded from Ensembl (www.ensembl.org, and those containing a full-length ORF were shuffled 50 times using the program CodonShuffle. CodonShuffle randomizes the nucleotide sequence by swapping synonomous codons, preserving the encoded amino acid sequence, codon usage, and base composition of the native mRNA [32]. Consequently, the program controls for constraints on the protein coding function of the mRNA, and for constraints on codon usage. Since the ORF is preserved by this type of shuffling, codon arrangements forbid the UAG portion of the UAGG motif to occur in-frame. The occurrence of UAGG was reduced by 1.5-fold in authentic coding sequences as compared to CodonShuffled control sequences (*p* < 0.001). Thus, the correlation of the motif with exon skipping is statistically significant, and there is modest selection against UAGG sequences for constitutive exons. Next we asked whether UAGG is overrepresented in skipped human exons. As expected, both UAGG and GGGG were found to be significantly overrepresented in skipped exons as compared to constitutive exons in human (χ^2^ = 436 and 87, respectively; *p* < 10^−5^ for both).

More rigorously, when all possible 5-mers were examined for overrepresentation in orthologous exons that are skipped in both human and mouse, a significant enrichment for U AGGC and U AGGG motifs was found (χ^2^ = 15 and 13, respectively; *p* < 10^−4^) compared to orthologous pairs of constitutive exons. U AGGA and UAGGU were not significantly overrepresented, but this may be explained by the small dataset used for the analysis (approximately 240 exons), or to functional overlap with ESE sequences. Nonetheless, the appearance of the UAGG motif in two 5-mers indicates the importance of the motif in conserved skipped exons. Overrepresentation of UAGG in skipped exons has also been found for mRNAs expressed in brain and testes, which are enriched for regulated splicing events [33].

### Identification of Skipped Exons with Conserved UAGG and GGGG Motif Patterns across the Human and Mouse Genomes

To identify exons unrelated to the CI cassette that might be silenced by a similar motif configuration, we focused in more detail on the UAGG and GGGG motif pattern by searching for these motifs singly and in combination in the database of approximately 96,000 human and mouse orthologous exons. Exons containing a GGGG in bases 3–10 of the intron and one or more exonic UAGGs were identified in the human and mouse subsets of the database and at the intersection of these datasets. These data are presented as Venn diagrams, and specific examples selected from the intersection dataset are shown to illustrate the motif patterns that are conserved in human and mouse orthologous exons (Figure 5). We included in the intersection dataset only exons in which the motif pattern is conserved in sequence and position in the human and mouse orthologous exons.

**Figure 5:**
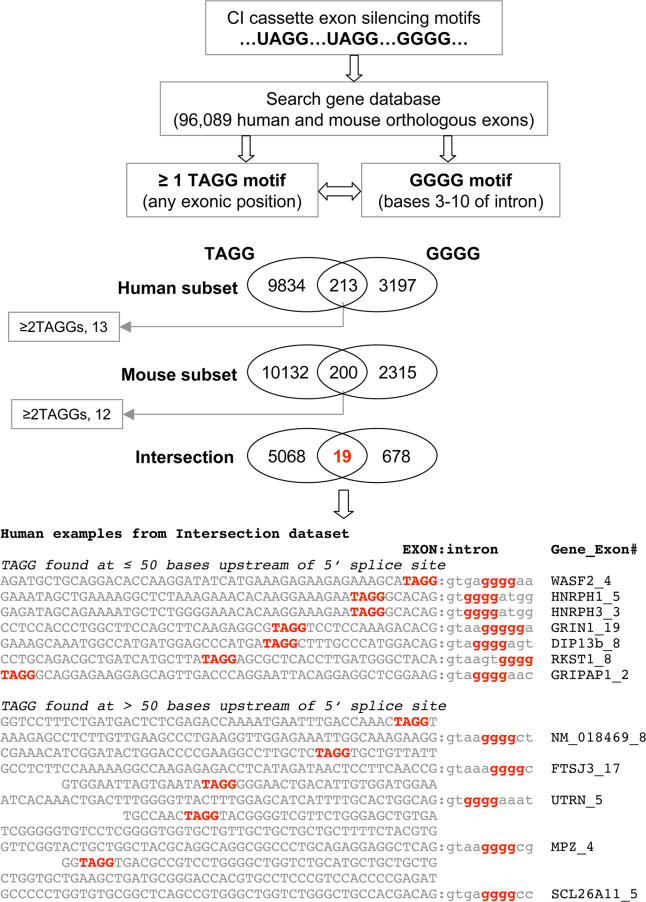
Genome-Wide Identification of Exons with UAGG and GGGG Silencing Motifs A database of 96,089 orthologous human and mouse exon pairs was searched for TAGG located anywhere in the exon and GGGG in bases 3–10 of the intron. Venn diagrams indicate the number of exons containing either or both sequence motifs in the human subset and the mouse subset of the database. The number of exons (19) in which UAGG and GGGG silencer motifs are conserved in orthologous human and mouse exons is also shown (intersection). The motif patterns are shown in the context of the exon (uppercase) and 5′ splice site region (lowercase) for 12 examples from the intersection dataset (human sequences are shown). Colon indicates 5′ splice site. The conserved TAGG and GGGG motifs are highlighted in red to illustrate variations in their positions. Gene name (HUGO ID) and exon number within the gene are indicated at far right. For one uncharacterized transcript, the GenBank accession is given instead (NM_018469_8).

As expected, the CI cassette exon of the *GRIN1* gene was found in all three of the overlap datasets. Of the 19 exons containing the motif pattern in the intersection dataset, 16 exons of 250 or fewer bases in length were considered for further study based on the observation that skipping of longer exons is quite rare [34]. This dataset contained the genes for two well known splicing factors, hnRNP H1 and H3 *(HNRPH1* and *HNRPH3).* Although human hnRNP H1 contains 14 exons and H3 contains ten exons, the UAGG and GGGG motif pattern was found associated with a single exon in each of these genes. As hnRNP H proteins are known to bind to guanosine-rich sequences, the presence of a conserved GGGG motif in the 5′ splice sites of these hnRNP H exons suggests the possibility of autoregulation at the level of splicing.

The hnRNP H exons and additional candidates in the intersection dataset (total of 12) were selected for experimental analysis of splicing patterns by RT-PCR, and to investigate the tissue specificity of the splicing patterns in human tissues (Figure 6; Table 1). The CI cassette exon was included in the analysis as a positive control *(GRIN1).* Skipping of the candidate exons for both human *HNRPH1* and *HNRPH3* was confirmed in several tissues. Exon skipping was also confirmed for candidate exons of *UTRN* and an uncharacterized hypothalamus-expressed gene, and tissue-specific exon skipping was evident for *HNRPH1* exon 5, *HNRPH3* exon 3, and *UTRN* exon 5. To our knowledge, these tissue-specific patterns have not been characterized previously. The results of Figure 6 were confirmed by DNA sequence analysis of the gel-purified products of the RT-PCR reactions. Although the candidate exon in the *ANXA8* gene was not experimentally validated in our analysis, EST and mRNA evidence confirms that the exon is skipped in cDNA libraries derived from choriocarcinomas (Table 1). An important caveat is that the true number of skipped exons could be significantly higher than that confirmed by RT-PCR because our sampling of human tissues in these experiments was not exhaustive.

**Figure 6:**
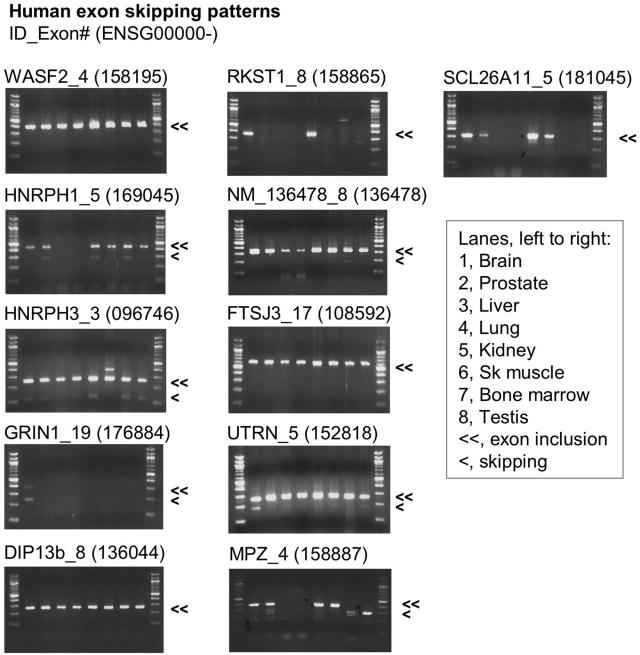
RT-PCR Confirmation of Exon Skipping Patterns in Human Tissues Eleven orthologous exons (≤250 nucleotides in length) were selected from the analysis of Figure 5 for RT-PCR analysis in a panel of eight human tissues. These exons are derived from the intersection dataset, in which conserved TAGG and GGGG motifs are present in combination in the human and mouse orthologous exons. Additional cDNA and EST evidence for these skipping events are summarized in Table 1. Specific primer pairs were designed for each test exon to amplify the exon-included (double arrowhead) and exon-skipped (single arrowhead) products by RT-PCR. Each gel panel shows the products of reactions for a single test exon resolved on agarose gels in the arrangement given in the inset. Gene name, exon number, and Ensembl number (in parentheses) are provided above each gel panel. The far left and far right lanes of each gel panel contain DNA molecular weight markers.

**Table 1:**
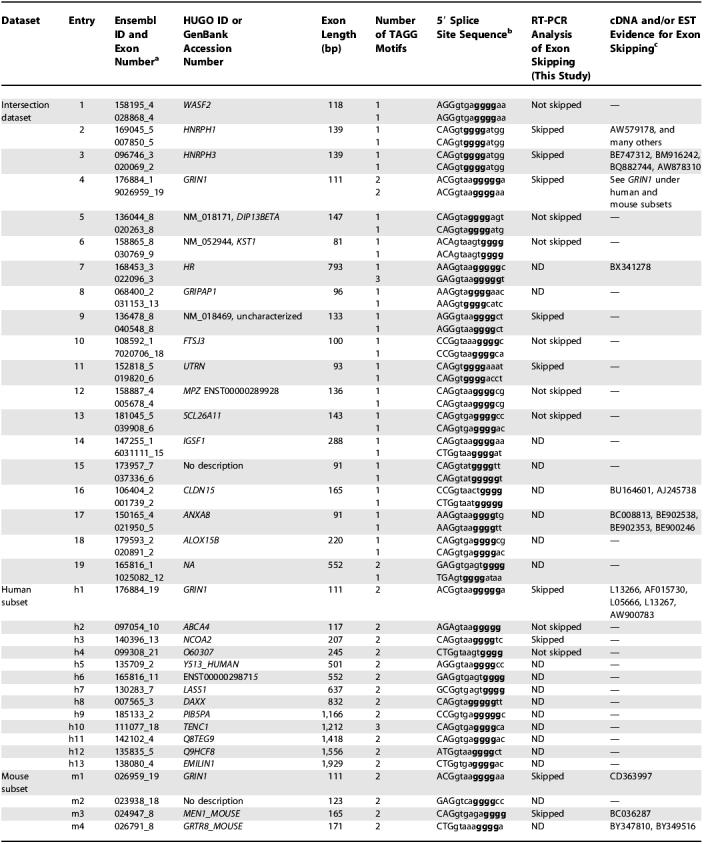
Human and Mouse Orthologous Exons Containing TAGG and GGGG Motif Patterns

Entries 1–19 correspond to the intersection dataset of Figure 5A, with the human exon listed above the mouse exon. The human subset, h1–h13, and mouse subset, m1–m12, are also shown. ^a^ The Ensembl ID prefixes are ENSG00000- for human and ENSMUSG00000- for mouse. ^b^ Uppercase indicates exonic and lowercase indicates intronic nucleotides. ^c^ Sources: http://genome.ucsc.edu and http://www.ncbi.nlm.nih.gov/.ND, not determined. DOI: 10.1371/journal.pbio.0030158.t001

**Table 1:**
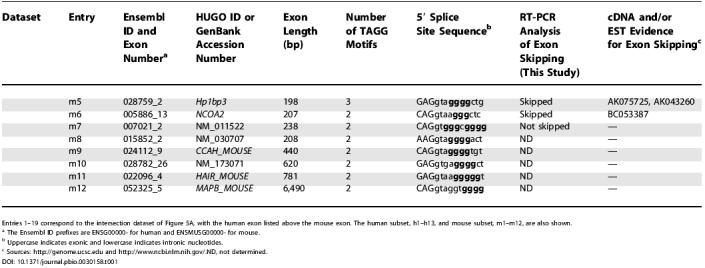
Continued

The mouse orthologs of *HNRPH1* exon 5 and *HNRPH3* exon 3 were chosen for further analysis of their splicing patterns (Figure 7, “1 TAGG + GGGG exons”). These splicing patterns were determined using RNA derived from mouse heart and brain tissue, as well as from the mouse C2C12 cell line. For each RNA sample, radioactive RT-PCR reactions were performed for a set of three serial dilutions of the input RNA. Good consistency in the percent exon inclusion values for each set of serial dilutions was evident. Sequence alignments showed that exon 3 of both the human and mouse *HNRPH3* genes contained an additional exonic GGGG motif not found in the orthologous *HNRPH1* exon 5 sequences (Figure 7, bottom), which might explain the higher rate of exon skipping observed. *HNRPH1* exon 8 and β-actin exon 2 served as control exons, since these exons do not contain UAGG or GGGG motifs (Figure 7, “0 TAGG, 0 GGGG exons”). As expected, the “0 TAGG, 0 GGGG” control exons showed 100% exon inclusion in each case.

**Figure 7:**
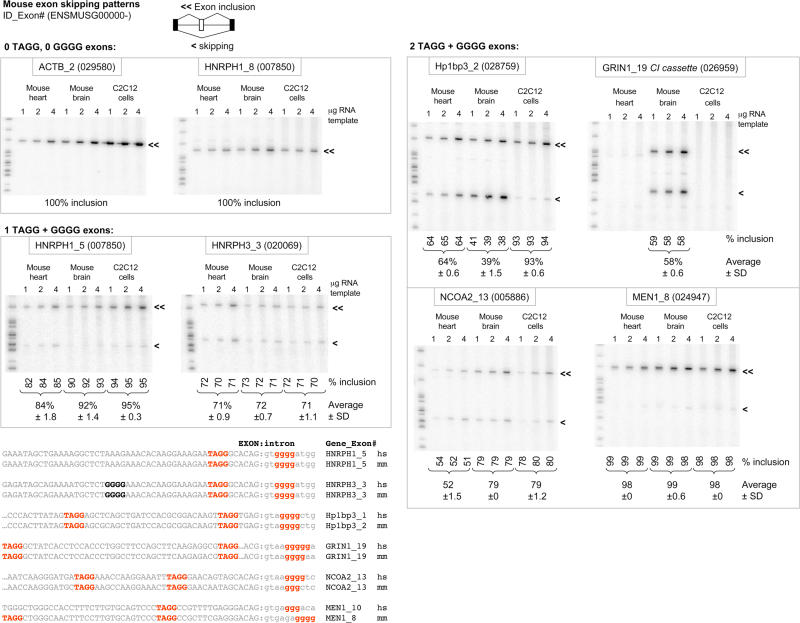
Analysis of Splicing Patterns in Mouse Tissues for Variations in the Number of Exonic UAGGs Splicing patterns were determined by radiolabeled RT-PCR for selected mouse exons. Control reactions include *β-actin* exon 2 and *HNRPH1* exon 8, which were selected because they lack the silencing motifs studied (“0 TAGG, 0 GGGG exons”). *HNRPH1* exon 5 and *HNRPH3* exon 3 are representative of the one TAGG plus GGGG motif pattern (“1 TAGG + GGGG exons”). *Hp1bp3* exon 2, *GRIN1* CI cassette exon, and *NCOA2* exon 13 are examples of tissue-specific exon skipping associated with the two TAGG plus GGGG motif pattern (“2 TAGG + GGGG exons”). *MEN1* exon 8 is also shown. Each gel panel shows splicing patterns tested in RNA samples from mouse heart and brain tissue and mouse C2C12 cells. Gene name, exon number, and Ensembl gene ID (in parentheses) are provided above each gel panel. Curly brackets point to the average percent exon inclusion and standard deviation for each set of serial dilutions; raw values are given immediately below each lane. Sequence alignments (bottom left) of the corresponding human and mouse orthologs illustrate the patterns of silencer motifs (orange). Bold indicates an additional exonic GGGG motif.

The observation that multiple UAGGs are associated with an increased strength of splicing silencing of the CI cassette exon (see Figure 2) prompted us to examine several exons with these characteristics that were identified in our searches. From the dataset of 213 human exons containing UAGG and GGGG, 13 exons with two or more UAGGs were identified, and from the dataset of 200 mouse exons containing UAGG and GGGG, 12 exons with two or more UAGGs were identified (Table 1). Exons within these datasets that had lengths typical for internal coding exons (≤250 bases) were chosen for RT-PCR analysis of their splicing patterns. RNA derived from mouse heart and brain and C2C12 cells confirmed the skipping of *Hp1bp3* exon 2 and *NCOA2* exon 13 and trace levels of skipping for *MEN1* exon 8 (see Figure 7). Additional cDNA evidence was found in the databases in support of these splicing patterns (Table 1). In the case of *Hp1bp3,* sequence alignments showed that two TAGGs and the 5′ splice site GGGG motif were conserved in the human and mouse orthologs, but these exons were not found in the intersection dataset of Figure 5 because the human exon corresponds to the first exon in the transcript, and consequently was not annotated as an internal exon in the Ensembl dataset. Sequence alignments for the more weakly skipped exons, *NCOA2* exon 13 and *MEN1* exon 8, showed that one or more segments of the motif pattern was imperfect in each set of orthologs (see Figure 7, bottom).

### Generality of the UAGG and GGGG Motif Pattern for Exon Silencing and Differential Regulation by hnRNP Proteins

To test whether the silencing motif pattern identified above for the CI cassette exon is sufficient for exon silencing in vivo, this pattern was introduced into the middle exon of a heterologous splicing reporter, *SIRT1* (Figure 8). This middle exon corresponds to the constitutively spliced exon 6 of the human *SIRT1* gene, and lacks any features of the silencing motif pattern to be examined. In these experiments the generality of the motif pattern, as well as the regulatory roles of hnRNP A1 and H were tested. When the GGGG motif was introduced by itself at intron positions 6–9 or 8–11 of the *SIRT1* splicing reporter (substrates *SIRT1*-G6–9 and *SIRT1*-G8–11, respectively), no change in the splicing pattern was observed relative to the parent substrate *SIRT1* (Figure 8, lanes 1, 4, and 7). These results indicate that in the *SIRT1* context, the GGGG motif alone is not sufficient to induce exon skipping. However, when two UAGGs were introduced into the middle exon (ESS19), the splicing pattern was shifted substantially, from 100% to 29% exon inclusion (Figure 8, lane 10). When the GGGG motif was subsequently introduced into the ESS19 substrate at intron positions 6–9 (ESS19-G6–9), exon inclusion was further reduced to 18% (Figure 8, lane 13), showing the combined effects of the motif pattern. In this context, the effect of the intronic GGGG motif was position dependent, since no additional silencing was observed when the GGGG was moved to positions 8–11 of the intron (ESS19-G8–11).

**Figure 8:**
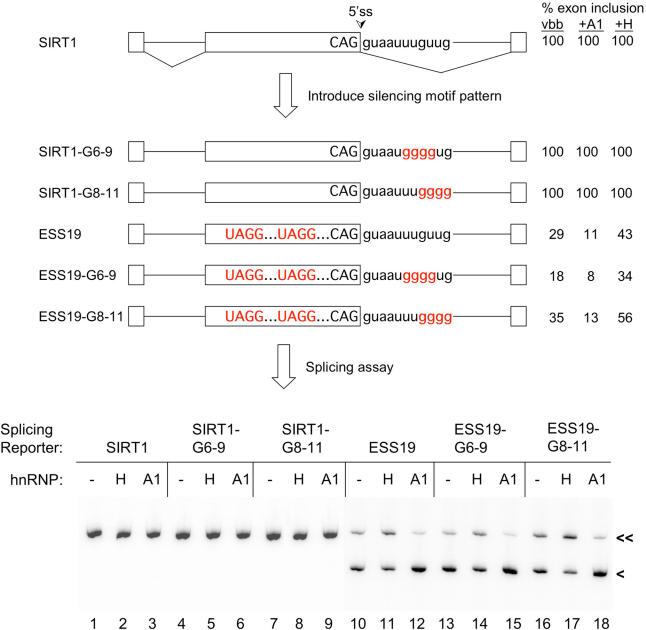
Analysis of UAGG and GGGG Motif Pattern in a Heterologous Context and Effects of hnRNP A1 and H Co-Expression At the top is a schematic of the heterologous splicing reporter *SIRT1* (pZW8) that contains exon 6 of the human *SIRT1* gene and flanking intron sequences as described previously [17]. The intron/exon lengths (in nucleotides) are as follows: exon 1, 308; intron 1, 340; exon 2, 95; intron 2, 287; and exon 3, 436. The silencing motif pattern was introduced sequentially into the middle exon and adjacent 5′ splice site region as highlighted in red. GGGG mutations were introduced by site-directed mutagenesis at positions 6–9 or 8–11 of the second intron. Exonic UAGG motifs were introduced into the middle exon by replacing a HindIII-KpnI restriction fragment AAGCTT TCGAATTCGGTACC, with AAGCTT GT **TAGG**TA TAGG TACC (restriction sites are underscored) as described [17]. The percent exon inclusion values (average of three repeats) were determined from co-expression assays with vector backbone (vbb) or with a 1:4 ratio of hnRNP A1 or H expression plasmid (same as experiment of Figure 3). Below are shown splicing assays following expression in C2C12 cells in the absence and presence of hnRNP A1 (“A1” lanes) or hnRNP H (“H” lanes) protein expression vector. Control reactions contained vector backbone plasmid (“−” lanes). Exon-included (double arrowheads) and exon-skipped (single arrowheads) products are indicated.

Based on the effects of hnRNP A1 and hnRNP H on the level of CI cassette exon inclusion described above (see Figure 3), we also tested the effects of these factors with the new splicing reporter substrates in co-expression assays. Relative to the vector backbone controls, the co-expression of hnRNP A1 down-regulated exon inclusion, consistent with the presence of the complete silencing motif pattern or exonic UAGGs, and co-expression of hnRNP H had the opposite effect (see Figure 8, lanes 10–18). The differential effects of hnRNP A1 and H were both dependent upon the presence of exonic UAGGs, since no change in the splicing pattern was observed for substrates *SIRT1, SIRT1*-G6–9, or *SIRT1*-G8–11 (see Figure 8, lanes 1–9). Interestingly, these results suggest that hnRNP H can exert its anti-silencing effect through the exonic UAGGs.

To further investigate the generality of exon silencing by UAGG and GGGG motifs, we examined a subset of the exons identified by bioinformatics to assess their splicing patterns and sensitivity to regulation by hnRNP A1 and hnRNP H in the *SIRT1* heterologous context. Exons containing the silencing motif pattern should be skipped exons, and regulation by these splicing factors would generally be expected for exons that contain the silencing motif pattern. For the convenience of testing new exons in this context, the *SIRT1* splicing reporter was modified to introduce restriction sites 12 nucleotides upstream and 12 nucleotides downstream of the middle exon. Test exons with 12 nucleotides of flanking intron on each side were then cloned from mouse genomic DNA and inserted in place of the *SIRT1* exon 6 between the restriction sites (Figure 9). As controls, the middle exon of the *SIRT1* splicing reporter and the middle exon of ESS19 were reinserted in this context to generate new splicing reporters identical to those tested above except for the added restriction sites. The splicing patterns of these modified substrates, *SIRT1*a and ESS19a, were found to be essentially identical to those of *SIRT1* and ESS19 shown above, which shows that the restriction sites have no effect on the splicing pattern in these assays.

**Figure 9:**
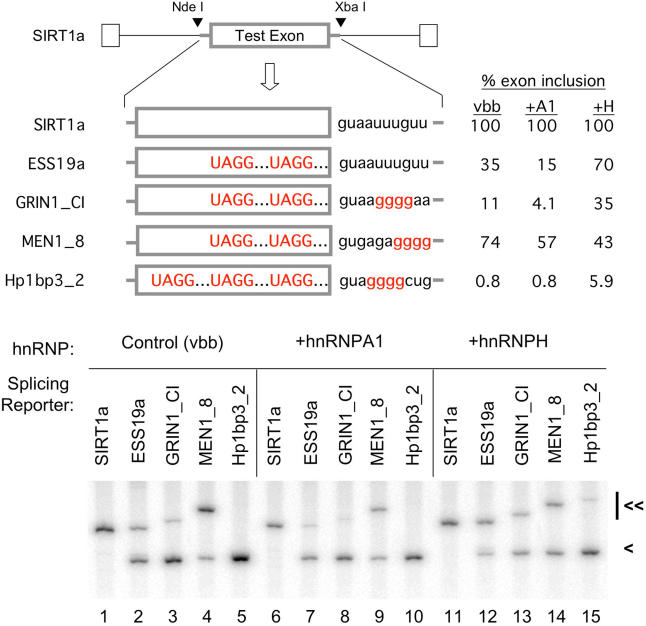
Exons Identified by Bioinformatics to Contain UAGG and GGGG Motif Patterns: Analysis of Splicing Patterns in a Heterologous Context and Effects of hnRNP Co-Expression Exons were cloned from mouse genomic DNA with 12 nucleotides of flanking intron sequence on each side. Each fragment was inserted into the *SIRT1*a context between the NdeI and XbaI restriction sites (Test Exon). *SIRT1*a is identical to *SIRT1* except for the NdeI and XbaI sites located 12 nucleotides upstream and downstream of the middle exon, respectively. The segment between the NdeI and XbaI restriction sites represents the region of *SIRT1*a replaced by test exons. ESS19a is the same as ESS19 except for the presence of the indicated restriction sites. Test exons included the CI cassette exon of rat *GRIN1* (GRIN1_CI), exon 8 of *MEN1* (MEN1_8), and exon 2 of *Hp1bp3* (Hp1bp3_2). Splicing reporters were expressed in C2C12 cells in the presence of vector backbone (vbb), or with hnRNPA1 or hnRNPH protein expression vector at a ratio of 1:4. Exon-included (double arrowheads) and exon-skipped (single arrowheads) mRNA products were quantified from the gel shown, and used to calculate the percent exon included values (top right).

Next we replaced the test exon of *SIRT1*a with the CI cassette exon of the rat *GRIN1* (*GRIN1*_CI), exon 8 of *MEN1* (*MEN1*_8), and exon 2 of *Hp1bp3* (*Hp1bp3*_2). In the absence of protein co-expression, exon skipping was observed in every case, although the extent of skipping varied over a wide range (Figure 9, “Control [vbb]”). For the CI cassette exon, hnRNP A1 induced 2.7-fold more skipping, whereas hnRNP H induced 3.2-fold more exon inclusion compared to the control sample (Figure 9, compare lanes 3, 8, and 13). Co-expression of hnRNP H increased the inclusion of exon 2 of *Hp1bp3* by a factor of 7.4, but no effects of hnRNP A1 were observed (Figure 9, lanes 5, 10, and 15). The latter may have been precluded by the extreme skipping pattern of this exon (0.8% inclusion), which contains three exonic UAGG motifs and a GGGG motif in the 5′ splice site. Thus, for these three exons, the regulation mediated by these hnRNP proteins is specified locally—that is, by sequences limited to the exon and adjacent splice sites. We cannot rule out the possible contributing roles of unknown sequence control elements in splicing silencing. However, sequence alignments show that these exons are highly diverse, and lack shared sequences longer than a few bases.

Co-expression of hnRNP A1 and hnRNP H was also observed to regulate exon 8 of *MEN1,* but with different results. Whereas exon skipping decreased as expected in the presence of hnRNP A1 (74% to 57% exon inclusion), exon skipping decreased to an even greater extent in the presence of hnRNP H (43% exon inclusion), indicating that both of these factors can silence the exon (Figure 9, lanes 4, 9, and 14). Because the *MEN1* exon contains two guanosine-rich ASF/SF2 motifs, 5′- GGGAGGA3′ and 5′- AGGAGGG-3′, capable of binding hnRNP H, the observed silencing effect of hnRNP H in this case is not surprising, and is likely explained by the disruption of exon enhancement.

Finally, the results observed for the ESS19 splicing reporter prompted another computational search to determine whether exon skipping is associated with two or more exonic UAGGs genome-wide. Similar to the analysis of Figure 4, exons containing two or more conserved UAGGs were identified from a large database (>94,000) of human and mouse exons and the cDNA/EST-confirmed skipped exons in that group were determined. From this analysis 163 human exons were found to contain two or more exonic UAGGs that are conserved in sequence and position in the orthologous mouse exons, and 16 of these (9.8%) were confirmed skipped exons (Figure 10). This was a significant enrichment of exon skipping (*p* < 0.002) compared to the remaining exons (90,028) lacking UAGGs, of which 4,160 (4.6%) were confirmed skipped exons. When the analysis was repeated for a single UAGG in the exon, a larger number of exons was identified (3,602), but a smaller percentage of confirmed skipped exons, 229 (6.4%), was associated with this group (*p* < 0.002). The list of 16 human exons with two or more conserved UAGGs and transcript evidence for skipping is shown in Figure 10, since these are novel candidates for alternative splicing regulation. Of particular interest are Elongator protein 2, NCOA2, Pumilio homolog 2, and RNA binding protein S1, which are implicated in RNA metabolism.

**Figure 10:**
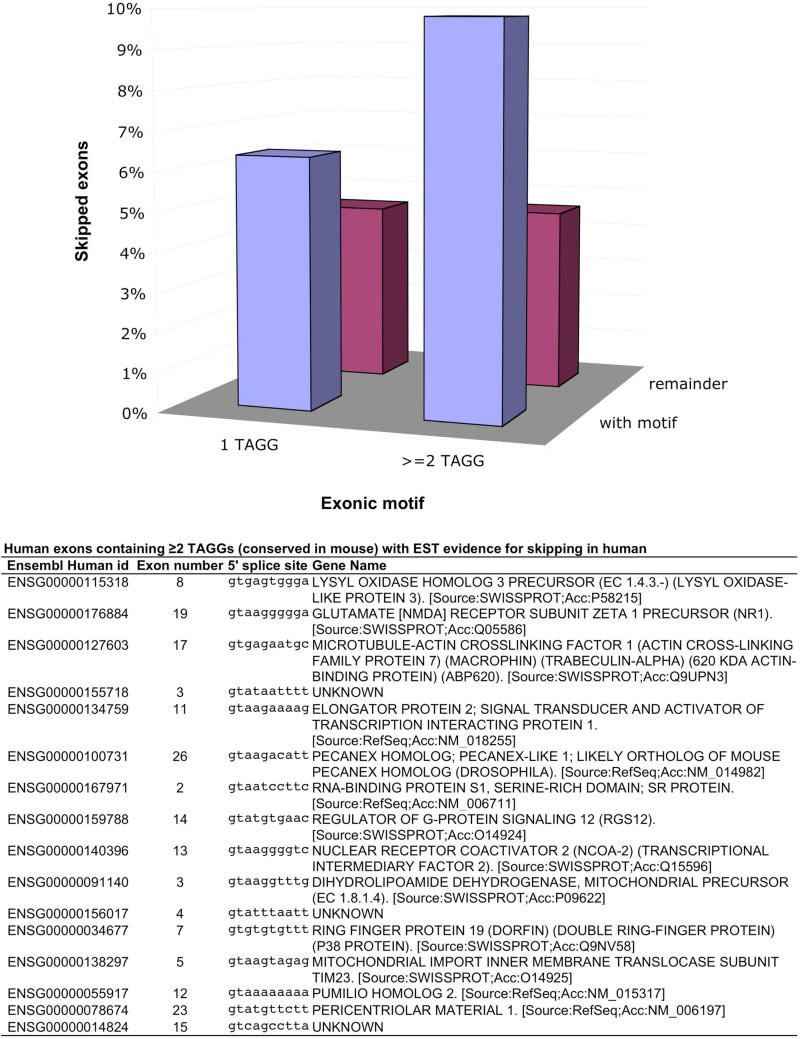
Computational Analysis of Exonic UAGG Motifs and Exon Skipping Patterns Genome-Wide Computational searches were performed to identify exons with two or more UAGGs and to determine the association of confirmed exon skipping events with this group. Exons with a single UAGG were analyzed for comparison. The following constraints were applied: (1) exon lengths of 250 bases or fewer and (2) both UAGG motifs conserved in sequence and position in the orthologous mouse exons. The graph illustrates the percentage of confirmed exon skipping events associated with one UAGG or two or more UAGGs (blue bars), or with the remaining exons lacking these motifs (red bars). The list of 16 human exons identified with two or more UAGGs is shown with the Ensembl ID, exon number, 5′ splice site sequence, and gene name. It is not unexpected to find exon 19 of the glutamate NMDA receptor *GRIN1* and exon 13 of *NCOA2,* which have a 5′-splice-site-proximal GGGG, since the sequence of the 5′ splice site was not specified in the search.

## Discussion

### A Combinatorial Code for Exon Silencing

Here we use molecular approaches to define a ternary combination of UAGG and GGGG motifs required for silencing the *GRIN1* CI cassette exon, and show that a class of skipped exons in the human and mouse genomes can be identified through bioinformatics searches that maintain the sequence and spatial configuration of the silencing motifs. We also illustrate, using the CI cassette model system, how the combined sequence motifs work cooperatively to determine the strength of exon silencing, with similar trends in neuronal and non-neuronal cell types. While a single exonic UAGG or 5′-splice-site-proximal GGGG motif specifies weak exon skipping, multiple UAGGs in the exon together with the GGGG motif at the 5′ splice site specifies predominant exon skipping (see Figure 2). This conclusion is strengthened by the complementary results observed when these RNA signals are systematically disrupted in a skipped exon or introduced into a constitutive exon. The CI cassette exon is converted into a constitutive exon by interrupting all three components of the motif pattern, whereas strong exon skipping results when the same components are introduced into constitutive exon 6 of the human *SIRT1* splicing reporter. In both contexts, hnRNP A1 co-expression mediates silencing and hnRNP H mediates anti-silencing in concert with all three components of the motif pattern (Figure 11).

**Figure 11:**
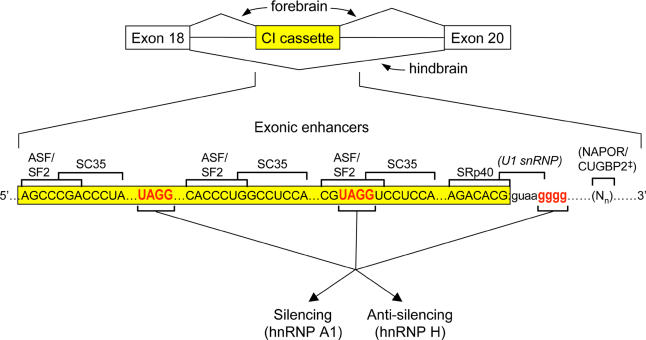
Model for Differential Regulation of the CI Cassette Exon by the Interplay of hnRNP A1 and H and a Ternary Motif Pattern At the top is a schematic of intron/exon structure and prominent splicing patterns observed in the forebrain (top) and hindbrain (bottom) of rat brain. Below is a summary of splicing regulatory motifs functionally defined in this study depicted on an expanded version of the *GRIN1* CI cassette exon (yellow). ESEs are indicated above the exon. Nucleotides complementary to U1 small nuclear RNA and the interaction of the positive regulator NAPOR/CUGBP2 with the downstream intron are indicated (‡; as determined in [26]). UAGG and GGGG splicing silencing motifs defined in this study are highlighted in red. The working model for splicing silencing, based on the results shown here, proposes that the CI cassette is a strong exon silenced by a combination of two exonic UAGG motifs and a 5′-splice-site-proximal GGGG. HnRNP A1 mediates silencing and hnRNP H mediates anti-silencing via these RNA signals.

In this study, bioinformatics searches show that the combination of exonic UAGG and 5′-splice-site-proximal GGGG motifs is relatively rare, since only 0.2% of a large database of human and mouse exons (approximately 200 out of approximately 96,000) harbor UAGG and GGGG motifs together in the correct arrangement. Nonetheless, based on cDNA and EST evidence a significantly higher frequency of exon skipping is associated with the set of 16 exons in which the motif pattern (≥1 exonic UAGGs and a 5′-splice-site-proximal GGGG) is conserved in the human and mouse orthologs (see Figure 4). For 14 of the newly identified exons we experimentally determined a rate of approximately 57% exon skipping based on RT-PCR analysis in a variety of human and mouse tissues (eight of 14, not counting the CI cassette). We would expect an imperfect correlation between the presence of the motif pattern and confirmed exon skipping, since the approximately 8–10 exonic enhancer motifs in a typical 140 base exon [13,15] may override the effects of UAGG and GGGG silencer motifs. This may be due not only to the arrangement of ESE and intronic splicing enhancer motifs in and around a target exon, but also to tissue-specific variations in splicing factors. Evidence was also shown for an increased association of confirmed exon skipping events genome-wide with the presence of two or more conserved exonic UAGGs, as a variation of the original motif pattern. Our functional analysis showed that the presence of multiple UAGGs in the same exon was an important parameter for a predominant exon skipping pattern.

The question of the relative 5′ splice site strengths of those exons containing or lacking the UAGG and GGGG motif pattern was also addressed. When the relative splice site strengths of the two groups were compared using a rank sum statistical test, no significant difference in the distributions was found. In fact the median score for splice site strength was found to be higher (9.31) for the group of exons containing the motifs than for those without (8.68). The close proximity of the motifs to, or their overlap with, the 5′ splice site, however, remains an unresolved issue. While a detectable effect of the (intronic) position of the GGGG motif was observed in the context of the *SIRT1* splicing reporter, the general rules for such position effects were not determined. GGGG or UAGG motifs in the 5′ splice site region may interfere with base pairing interactions involving U1 and/or U6 small nuclear RNAs, and these effects may have a high degree of position dependence.

Numerous ESE motifs have been functionally identified in concert with the regulatory roles of SR proteins, but far less is known about sequence motifs and factors that control silencing. Evidence for exonic UAG and UAGG motifs has been previously reported for splicing silencing mechanisms mediated by hnRNP A1. These include the K-SAM exon of human *FGFR2* [35], *SMN2* exon 7 (U AGACA) [36], HIV *Tat* exon 2 (U AGACU) [37,38], *CD44* exon v5 (U AGACA) [39], *protein 4.1* exon 16 [40], *c-src* exon N1 (UAG: GAGGAAGGU) [41], and exons in the hnRNP A1 transcript itself (UAG and U AGAGU) [24,42]. Taken together with structural evidence that hnRNP A1 recognizes TAGG motifs directly [43], A1 is a likely mediator of many if not all of these silencing events. In contrast to the previous studies, however, the 5′-splice-site-proximal GGGG motif is a novel and integral component of the silencing mechanism of the CI cassette exon. While the silencing effect of the GGGG motif by itself is slight, its function with exonic UAGGs is synergistic. Our computational analysis using the CodonShuffle algorithm extends these previous studies by showing genome-wide that the UAGG motif is significantly underrepresented in constitutive exons and overrepresented in skipped exons. Because the CodonShuffle analysis forbids in-frame UAG stop codons, these results are in good agreement with the idea that exonic UAGG motifs function widely as splicing silencers.

In previous studies guanosine-rich motifs have been shown to regulate splicing in diverse ways. Guanosine triplets are generally enriched in short mammalian introns [44,45], and these sequences have been shown to enhance inclusion of an unusually small exon of *cardiac troponin T* [46,47], as well as additional exons of human *α-globin* [48] and chicken *β-tropomyosin* [49], transcripts. Moreover, a disease-related point mutation in a guanosine cluster at position 26 of the intron has been shown to disrupt the normal pattern of splicing of the human *pyruvate dehydrogenase E1α* transcript [50]. In some cases, hnRNP H has been implicated in splicing control together with guanosine-rich sequences. A guanosine-rich ESS in *β-tropomyosin* exon 7 is required for exon skipping, and the degree of hnRNP H binding correlates with exon 7 skipping [51]. The *c-src* transcript contains a complex intronic enhancer downstream of the neuron-specific NI exon in which multiple guanosine-rich tracts are found that bind to hnRNP H and F and that are required for normal patterns of NI exon inclusion [52,53,54]. In addition, hnRNP H has been shown to bind to the 5′ splice site of *NF-1* exon 3, where it is thought to induce exon skipping when the splice site is weakened by a guanosine to cytosine mutation at position +5 of the intron [55]. In this study we show that hnRNP H has a positive effect on exon inclusion for three unrelated exons harboring the UAGG and GGGG motif pattern in the context of a heterologous splicing reporter. Because these exons have no other sequence relatedness, these results suggest that antagonism with hnRNP A1 might be a frequent property of hnRNP H in this type of silencing mechanism (see Figure 9).

### Model for Splicing Regulation Mediated by a UAGG and GGGG Code: Differential Roles of hnRNP A1 and H

A full understanding of CI cassette exon regulation will require explanations for the complex spatial and temporal variations observed in vivo. Based on functional evidence, we proposed in a previous study that NAPOR/CUGBP2 enhances CI exon inclusion in the rat forebrain, where its expression is enriched. It would be reasonable to predict, however, that the CI cassette exon is inherently a strong exon and should not require a positive regulator, since its splice sites match well to consensus sequences. Here we confirmed this prediction by experimental manipulations of the UAGG and GGGG motif pattern that converted the CI cassette exon into a constitutive exon in the absence of NAPOR/CUGBP2 (splicing reporter T8; see Figure 2). These results clearly demonstrate differential roles of hnRNP A1 and H proteins. Furthermore, we showed that the mechanism by which these proteins regulate the CI cassette can be controlled locally through sequences in the exon and adjacent 5′ splice site independent of any distal downstream intron sequences from the *GRIN1* transcript.

Six ESE motifs within the CI cassette exon were functionally identified in this study, and a seventh, an ASF/SF2 motif, overlaps with the exon position 93 UAGG silencer (see Figure 11). Predominant exon skipping was retained even when both of the natural UAGGs were carefully repositioned in the exon without destroying or creating any known ESEs. That is, in two distinct cell lines, the six functional ESE motifs did not overpower the silencing function of the three-part UAGG and GGGG code. We observed that UAGG motifs are embedded in 32 ESE motifs reported in the ESEFinder database [56], suggesting that the occurrence of overlapping ESE and ESS signals might be quite frequent. In the case of the CI cassette exon, such an arrangement of opposing splicing signals would predict that competition between ASF/SF2 and hnRNP A1 may provide additional options to fine-tune splicing patterns in different tissues or stages of development. However, in comparison to hnRNP H, the co-expression of an ASF/SF2 expression plasmid had only a mild positive effect on exon inclusion in the cell lines tested (K. H. and P. J. G., unpublished data).

Here we show evidence for combinatorial regulation by two different types of RNA elements (UAGG and GGGG) together with differential roles of hnRNP A1 and H (and F), but not all of the combinatorial interactions were experimentally defined. Although the intronic GGGG motif and A1 are involved in silencing, site-specific UV crosslinking of A1 to the GGGG motif was not observed (K. H. and P. J. G., unpublished data). This may be due to limitations of the assay, since UV crosslinking of A1 to its high-affinity site is inefficient [30]. Alternatively, the intronic GGGG may play a structural role, or contact an additional protein factor involved in the assembly of the putative silencing complex. We speculate that a silencing complex is formed by the interactions of hnRNP A1 monomers with individual UAGG and GGGG sites together with cooperative interactions between these monomers. We also speculate that hnRNP H and, to a lesser extent, F function principally as anti-silencing factors in the CI cassette mechanism by binding to the GGGG and/or UAGG motifs in a way that disrupts the cooperative binding of A1. In our view this is the simplest model to account for our experimental results, but more complex mechanisms cannot be ruled out at this point. Future studies will be required to establish how the various isoforms of hnRNP H carry out anti-silencing, and whether accessory factors are involved.

Substantial evidence exists in support of models involving competition between hnRNP A1 and SR proteins in modulating 5′ splice site selection or exon inclusion [24,57,58,59,60,61]. The involvement of hnRNP A1 in the CI cassette mechanism is also consistent with previous demonstrations of the cooperative binding of hnRNP A1 to pre-mRNAs [62,63,64,65]. Based on the analysis of microarray data [66,67] documenting considerable variations in the ratios of hnRNP A1 transcripts to hnRNP F and H transcripts in human and mouse [33], we suggest that such variations may be involved in directing tissue specificity of exons that are regulated by UAGG and GGGG motifs.

### Implications of Genome-Wide Analysis

Since the CI cassette exon skipping pattern of the *GRIN1* transcript is brain-region-specific, we wished to determine the splicing characteristics of other exons with a similar arrangement of these motifs in the human and mouse genomes. Other transcripts harboring skipped exons that were identified by bioinformatics searches, however, were found to be involved in a variety of cellular functions, such as RNA processing, chromatin structure/function, cell signaling, and regulation of transcription. These include hnRNP H1 and H3 *(HNRPH1* and *HNRPH3),* menin *(MEN1),* nuclear receptor co-activator 2 *(NCOA2),* heterochromatin protein 1 binding protein 3 *(Hp1bp3),* and an uncharacterized hypothalamus transcript (Table 1). A high proportion of the exon skipping patterns identified were found to be tissue-specific.

The observation that exon 5 of *HNRPH1* and exon 3 of *HNRPH3* contain conserved UAGG and GGGG motifs is intriguing, since hnRNP H proteins crosslink specifically to the GGGG motif adjacent to the CI cassette exon. These exon skipping patterns were confirmed by RT-PCR analysis in this study, and there is additional supporting cDNA and EST evidence in the databases. The RT-PCR analysis shows that these exon skipping patterns are relatively weak, but this is consistent with a motif pattern containing a single exonic UAGG and 5′ splice site GGGG motif. Skipping of exon 5 of *HNRPH1* or exon 3 of *HNRPH3* would result in a shift in the reading frame and introduction of a premature termination codon. Thus, silencing of these exons at the level of splicing is expected to reduce protein expression via either nonsense-mediated mRNA decay or premature termination of protein synthesis. The results shown here suggest a model in which hnRNP H proteins may provide a buffering effect against negative control by hnRNP A1. Autoregulation by a negative feedback loop was recently demonstrated for the splicing factor PTB, which induces skipping of the 11th exon of its cognate pre-mRNA [68]. Similarly, hnRNP A1, SRp20, SC35, TIA1, and TIAR proteins are all involved in mechanisms that regulate the splicing patterns of their cognate transcripts [69,70].

### Prospects

If alternative splicing events are as prevalent as recent studies suggest [21,22,71,72], it will be important to understand on a global scale the biochemical language that determines tissue-specific patterns, and tunes these patterns in response to physiological stimuli [73,74]. Here we show that UAGG and GGGG motifs function in combination to silence the CI cassette exon and also serve more generally as patterns to recognize other skipped exons in the human and mouse genomes. Combinatorial splicing control mechanisms are not well understood, and previous studies have not addressed the brain-region-specific splicing switch that is characteristic of the CI cassette exon. Our results suggest that, in general, it might be a useful strategy to use motif pattern searches, together with information about spatial constraints, to identify co-regulated exons. The observation that UAGG and GGGG motif patterns are generally predictive of exon skipping may also be useful in interpreting the effects of mutations underlying certain genetic diseases. Future work will be needed to more fully understand the roles of hnRNP proteins in this type of silencing (and anti-silencing) mechanism, and to further advance the understanding of the complex biochemical language responsible for the regulation and coordination of splicing events genome-wide.

## Materials and Methods

### 

#### Plasmid construction and mutagenesis.

All splicing reporter plasmids except for those in the experiments of Figure 3D were derived from the parent plasmid wt (previously called E21wt), in which the CI cassette exon is flanked by full-length introns and adjacent exons [26]. Site-directed mutations were introduced into the CI cassette exon or downstream intron using the QuikChange Site-Directed Mutagenesis Kit (Stratagene, La Jolla, California, United States), and mutations were confirmed by DNA sequencing. The splicing reporters wt and wt0 are identical except that wt has a point mutation at position 78 (C to G change) of the CI exon, which creates a XhoI site. Chimeric splicing reporters were derived from parent plasmid rGγ25 [75], in which the CI cassette exon and 164 and 103 bp of the flanking introns (upstream and downstream, respectively) were introduced as a NotI-BamHI fragment. The full-length upstream intron was introduced by replacing the XbaI-NotI fragment of rGγCI-wt0 with the XbaI-NotI PCR product containing *GRIN1* exon 18 and 1,092 bp of adjacent intron (plasmid, rGγCI-up). The full-length downstream intron was introduced by replacing the BamHI-EcoRI fragment of rGγCI-wt0 with the BamHI-EcoRI fragment containing *GRIN1* exon 20 and 1,810 bp of adjacent upstream intron. All splicing reporter plasmids were constructed in a pBS vector followed by transfer into the vector pBPSVPA+ [76], in which expression is driven by the SV40 promoter. Expression plasmids for hnRNP proteins F, H, and A1 were generated by subcloning the complete open reading frames into the BamHI site of pcDNA4/HisMax vector (Invitrogen, Carlsbad, California, United States). Open reading frames were obtained from the following plasmids: hnRNP F from plasmid pFlag-F [53], hnRNP H/H′ from pFlag-DSEF-1 [77,78], and hnRNP A1 from plasmid Myc-A1 [79]. All plasmid constructs were confirmed by DNA sequencing, and protein expression was verified by Western blot analysis.

#### Transient expression and analysis of RNA splicing patterns.

Growth of C2C12 cells, transfection, and RT-PCR analysis were performed as described [26]. Briefly, transfections were performed in 60-mm plates at approximately 70% cell confluency using Lipofectamine (Invitrogen). Transfections contained 3.5 μg of total plasmid DNA made up of splicing reporter plasmid with empty vector and/or protein expression plasmid at the DNA ratios specified. PC12 cells were grown to approximately 85% confluency in RPMI1640 supplemented with 10% fetal bovine serum and 5% horse serum on poly-D-lysine-coated six-well plates. PC12 cell transfections were carried out with Lipofectamine 2000 and a total of 1.25 μg of plasmid DNA (0.25 μg of splicing reporter and 1 μg of protein expression plasmid or vector backbone). After 48 h, cells (C2C12 and PC12) were harvested and total RNA was purified, DNase I treated, and ethanol precipitated. For analysis of splicing patterns, 1 μg of RNA was reverse transcribed with random hexanucleotide primers, and 1/20th of the reaction volume was then amplified for 20–24 PCR cycles in a 10-μl reaction containing 0.2 μM specific primers, two units of Taq polymerase, 0.2 mM dNTPs, and 1 μCi of [α^32^P]dCTP in reaction buffer. Under these conditions approximately 1% of the C residues in each product molecule are radiolabeled. Primers used to amplify the CI-cassette-exon-included and -skipped mRNA products were specific for the flanking exons. Sequences from Ensembl were used to design primers for the experiments of Figure 4. Primer sequences are available upon request. For gel analysis, 25% v/v of each PCR reaction was resolved on 6% polyacrylamide/5 M urea sequencing gels. Electrophoresis was performed for 1 h at 30 W. Gel images were obtained and results quantitated using a Fuji (Tokyo, Japan) Medical Systems BAS-2500 phosphorimager and Science 2003 ImageGauge software. For the experiment of Figure 6, PCR reaction products were resolved on 1% agarose gels in 1× TBE buffer.

#### Transcription and site-specific RNA labeling.

Radioactive RNA substrates were prepared for UV crosslinking analysis as follows. RNAs containing the GGGG motif were prepared by in vitro transcription in 25-μl reactions containing T7 RNA polymerase, 0.4 mM each of ATP, UTP, and CTP, and 0.3 mM GTP plus 25 μCi of [α^32^P]GTP, 0.5 mM GpppG, and 0.1 μg of DNA template in standard T7 reaction buffer. DNA templates were prepared by annealing complementary oligonucleotides with the top strand containing the T7 promoter sequence at its 5′ end, followed by the RNA test sequence; bottom strands were complementary to the test sequence. RNAs were purified after DNase treatment by Sephadex G25 chromatography, phenol extraction, and ethanol precipitation. Site-specific labeling of RNA substrates containing the exonic UAGG motif was performed essentially as described [80]. Transcription (nonradioactive) of the downstream RNA half was performed as above except that reactions were larger (125 μl) and contained 2 mM guanosine instead of GpppG. After gel purification, the 5′ end of the downstream-half RNA was labeled by polynucleotide kinase with 25 pmol of the purified RNA and 25 pmol of [γ^32^P]ATP (6,000 Ci/mmol). After removal of ATP by Sephadex G25 chromatography, the upstream and downstream RNA halves were annealed to a complementary DNA splint covering 16 bases on either side of the desired ligation position. Ligation reactions were performed in 10-μl reactions with 15 Weiss Units of T4 DNA ligase for 4 h at 16 °C, followed by DNase treatment and gel purification. The concentrations and integrity of the RNA preparations were verified by electrophoresis on 10% polyacrylamide/7M urea gels.

#### UV crosslinking and immunoprecipitation analysis.

UV crosslinking reactions (12.5 μl) were performed under splicing conditions as described [81] with 100,000 dpm radiolabeled RNA transcript and HeLa nuclear extract (4 mg/ml final concentration). Following UV treatment, samples were digested to completion with RNase A (1 mg/ml, 20 min at 30 °C), and held on ice for immunoprecipitation or SDS-PAGE analysis. For immunoprecipitation reactions, 25 μl of protein A beads (Sigma, St. Louis, Missouri, United States) were equilibrated in Buffer A (10 mM Tris/HCl [pH 7.5], 100 mM NaCl, and 1% TritonX100), and antibody was bound to the beads for 1 h on ice (5 μl of R7263 or R7264 for analysis of hnRNP F and H, respectively [82], or 1 μl of 9H10 for analysis of hnRNP A1). Equivalent concentrations of rabbit preimmune serum or purified mouse IgG were used for control reactions. Antibody beads were washed three times with Buffer A, and added to UV crosslinking reactions (25 μl) for 20 min on ice. Bound samples were washed four times with Buffer A, and centrifuged to separate pellet and supernatant. Each reaction component was boiled in SDS sample buffer, and resolved on discontinuous 12.5% polyacrylamide gels.

#### Generation of datasets and computational analysis.

Human and mouse genes that were annotated as orthologs were obtained from Ensembl release 16 (http://www.ensembl.org). Human–mouse exons were aligned by BLAST (requiring percent identity ≥85 and bit score ≥20), and genes were checked for consistency in terms of orthologous exon order. A total of approximately 94,000 conserved human–mouse exons were retained for further analysis (http://genes.mit.edu/burgelab/Supplementary/han04). In a separate analysis, approximately 14,600 internal exons from human genes were designated as skipped exons based on stringent alignments of cDNA and EST sequences to cDNA-verified genomic loci using the genome annotation script GENOA (http://genes.mit.edu/genoa). Mapping these exons to the conserved human–mouse Ensembl set identified 4,455 skipped internal human exons that are conserved in mouse. For the codon shuffling analysis, the first 30 bases and the last 60 bases of the original sequences were removed prior to shuffling to simulate removal of the first and last exons. Each sequence was shuffled 50 times using the CodonShuffle program [32]. The number of occurrences of each oligonucleotide, e.g., UAGG, divided by the number of occurrences of all possible oligonucleotides of equal length, was compared to the corresponding frequency of occurrence in the shuffled sets. The final fold underrepresentation was computed by taking the mean of the fractions computed over the shuffled sets, and dividing by the observed (true) fraction. The *p*-value for the reduced occurrence of UAGG in authentic coding sequences was determined by counting the number of 4-mers that were greater than 1.488-fold reduced relative to the average of 100 shuffles. None were found for each of the ten shuffles. Thus the *p*-value is 0/256, or *p* < 0.001.

## Abbreviations

ESE: exonic splicing enhancer
ESS: exonic splicing silencer
SR: serine-arginine-rich
hnRNP: heteronuclear ribonucleoprotein

## References

[pbio-0030158-b01] Lander ES, Linton LM, Birren B, Nusbaum C, Zody MC (2001). Initial sequencing and analysis of the human genome. Nature.

[pbio-0030158-b02] Maniatis T, Tasic B (2002). Alternative pre-mRNA splicing and proteome expansion in metazoans. Nature.

[pbio-0030158-b03] Wang HY, Xu X, Ding JH, Bermingham JR, Fu XD (2001). SC35 plays a role in T cell development and alternative splicing of CD45. Mol Cell.

[pbio-0030158-b04] Xie J, Black DL (2001). A CaMK IV responsive RNA element mediates depolarization-induced alternative splicing of ion channels. Nature.

[pbio-0030158-b05] Shin C, Feng Y, Manley JL (2004). Dephosphorylated SRp38 acts as a splicing repressor in response to heat shock. Nature.

[pbio-0030158-b06] Shin C, Manley JL (2002). The SR protein SRp38 represses splicing in M phase cells. Cell.

[pbio-0030158-b07] Caceres JF, Kornblihtt AR (2002). Alternative splicing: Multiple control mechanisms and involvement in human disease. Trends Genet.

[pbio-0030158-b08] Cartegni L, Chew SL, Krainer AR (2002). Listening to silence and understanding nonsense: Exonic mutations that affect splicing. Nat Rev Genet.

[pbio-0030158-b09] Faustino NA, Cooper TA (2003). Pre-mRNA splicing and human disease. Genes Dev.

[pbio-0030158-b10] Zhou Z, Licklider LJ, Gygi SP, Reed R (2002). Comprehensive proteomic analysis of the human spliceosome. Nature.

[pbio-0030158-b11] Makarov EM, Makarova OV, Urlaub H, Gentzel M, Will CL (2002). Small nuclear ribonucleoprotein remodeling during catalytic activation of the spliceosome. Science.

[pbio-0030158-b12] Jurica MS, Moore MJ (2003). Pre-mRNA splicing: Awash in a sea of proteins. Mol Cell.

[pbio-0030158-b13] Fairbrother WG, Yeh RF, Sharp PA, Burge CB (2002). Predictive identification of exonic splicing enhancers in human genes. Science.

[pbio-0030158-b14] Ladd AN, Cooper TA (2002). Finding signals that regulate alternative splicing in the post-genomic era. Genome Biol.

[pbio-0030158-b15] Zhang XH, Chasin LA (2004). Computational definition of sequence motifs governing constitutive exon splicing. Genes Dev.

[pbio-0030158-b16] Zheng ZM (2004). Regulation of alternative RNA splicing by exon definition and exon sequences in viral and mammalian gene expression. J Biomed Sci.

[pbio-0030158-b17] Wang Z, Rolish ME, Yeo G, Tung V, Mawson M (2004). Systematic identification and analysis of exonic splicing silencers. Cell.

[pbio-0030158-b18] Blencowe BJ (2000). Exonic splicing enhancers: Mechanism of action, diversity and role in human genetic diseases. Trends Biochem Sci.

[pbio-0030158-b19] Tacke R, Manley JL (1999). Determinants of SR protein specificity. Curr Opin Cell Biol.

[pbio-0030158-b20] Shen H, Green MR (2004). A pathway of sequential arginine-serine-rich domain-splicing signal interactions during mammalian spliceosome assembly. Mol Cell.

[pbio-0030158-b21] Modrek B, Resch A, Grasso C, Lee C (2001). Genome-wide detection of alternative splicing in expressed sequences of human genes. Nucleic Acids Res.

[pbio-0030158-b22] Johnson JM, Castle J, Garrett-Engele P, Kan Z, Loerch PM (2003). Genome-wide survey of human alternative pre-mRNA splicing with exon junction microarrays. Science.

[pbio-0030158-b23] Wagner EJ, Garcia-Blanco MA (2001). Polypyrimidine tract binding protein antagonizes exon definition. Mol Cell Biol.

[pbio-0030158-b24] Chabot B, LeBel C, Hutchison S, Nasim FH, Simard MJ (2003). Heterogeneous nuclear ribonucleoprotein particle A/B proteins and the control of alternative splicing of the mammalian heterogeneous nuclear ribonucleoprotein particle A1 pre-mRNA. Prog Mol Subcell Biol.

[pbio-0030158-b25] Wang Z, Grabowski PJ (1996). Cell- and stage-specific splicing events resolved in specialized neurons of the rat cerebellum. RNA.

[pbio-0030158-b26] Zhang W, Liu H, Han K, Grabowski PJ (2002). Region-specific alternative splicing in the nervous system: Implications for regulation by the RNA-binding protein NAPOR. RNA.

[pbio-0030158-b27] Ehlers MD, Tingley WG, Huganir RL (1995). Regulated subcellular distribution of the NR1 subunit of the NMDA receptor. Science.

[pbio-0030158-b28] Mu Y, Otsuka T, Horton AC, Scott DB, Ehlers MD (2003). Activity-dependent mRNA splicing controls ER export and synaptic delivery of NMDA receptors. Neuron.

[pbio-0030158-b29] Smith CW, Valcarcel J (2000). Alternative pre-mRNA splicing: The logic of combinatorial control. Trends Biochem Sci.

[pbio-0030158-b30] Burd CG, Dreyfuss G (1994). RNA binding specificity of hnRNP A1: Significance of hnRNP A1 high-affinity binding sites in pre-mRNA splicing. EMBO J.

[pbio-0030158-b31] Caputi M, Zahler AM (2001). Determination of the RNA binding specificity of the heterogeneous nuclear ribonucleoprotein (hnRNP) H/H′/F/2H9 family. J Biol Chem.

[pbio-0030158-b32] Katz L, Burge CB (2003). Widespread selection for local RNA secondary structure in coding regions of bacterial genes. Genome Res.

[pbio-0030158-b33] Yeo G, Holste D, Kreiman G, Burge CB (2004). Variation in alternative splicing across human tissues. Genome Biol.

[pbio-0030158-b34] Sorek R, Shamir R, Ast G (2004). How prevalent is functional alternative splicing in the human genome?. Trends Genet.

[pbio-0030158-b35] Del Gatto F, Gesnel MC, Breathnach R (1996). The exon sequence
TAGG can inhibit splicing. Nucleic Acids Res.

[pbio-0030158-b36] Kashima T, Manley JL (2003). A negative element in SMN2 exon 7 inhibits splicing in spinal muscular atrophy. Nat Genet.

[pbio-0030158-b37] Si Z, Amendt BA, Stoltzfus CM (1997). Splicing efficiency of human immunodeficiency virus type 1 tat RNA is determined by both a suboptimal 3′ splice site and a 10 nucleotide exon splicing silencer element located within tat exon 2. Nucleic Acids Res.

[pbio-0030158-b38] Bilodeau PS, Domsic JK, Mayeda A, Krainer AR, Stoltzfus CM (2001). RNA splicing at human immunodeficiency virus type 1 3′ splice site A2 is regulated by binding of hnRNP A/B proteins to an exonic splicing silencer element. J Virol.

[pbio-0030158-b39] Matter N, Marx M, Weg-Remers S, Ponta H, Herrlich P (2000). Heterogeneous ribonucleoprotein A1 is part of an exon-specific splice-silencing complex controlled by oncogenic signaling pathways. J Biol Chem.

[pbio-0030158-b40] Hou VC, Lersch R, Gee SL, Ponthier JL, Lo AJ (2002). Decrease in hnRNP A/B expression during erythropoiesis mediates a pre-mRNA splicing switch. EMBO J.

[pbio-0030158-b41] Rooke N, Markovtsov V, Cagavi E, Black DL (2003). Roles for SR proteins and hnRNP A1 in the regulation of c-src exon N1. Mol Cell Biol.

[pbio-0030158-b42] Chabot B, Blanchette M, Lapierre I, La Branche H (1997). An intron element modulating 5′ splice site selection in the hnRNP A1 pre-mRNA interacts with hnRNP A1. Mol Cell Biol.

[pbio-0030158-b43] Ding J, Hayashi MK, Zhang Y, Manche L, Krainer AR (1999). Crystal structure of the two-RRM domain of hnRNP A1 (UP1) complexed with single-stranded telomeric DNA. Genes Dev.

[pbio-0030158-b44] McCullough AJ, Berget SM (1997). G triplets located throughout a class of small vertebrate introns enforce intron borders and regulate splice site selection. Mol Cell Biol.

[pbio-0030158-b45] Lim LP, Burge CB (2001). A computational analysis of sequence features involved in recognition of short introns. Proc Natl Acad Sci U S A.

[pbio-0030158-b46] Carlo T, Sterner DA, Berget SM (1996). An intron splicing enhancer containing a G-rich repeat facilitates inclusion of a vertebrate micro-exon. RNA.

[pbio-0030158-b47] Carlo T, Sierra R, Berget SM (2000). A 5′ splice site-proximal enhancer binds SF1 and activates exon bridging of a microexon. Mol Cell Biol.

[pbio-0030158-b48] McCullough AJ, Berget SM (2000). An intronic splicing enhancer binds U1 snRNPs to enhance splicing and select 5′ splice sites. Mol Cell Biol.

[pbio-0030158-b49] Sirand-Pugnet P, Durosay P, Brody E, Marie J (1995). An intronic (A/U)GGG repeat enhances the splicing of an alternative intron of the chicken beta-tropomyosin pre-mRNA. Nucleic Acids Res.

[pbio-0030158-b50] Mine M, Brivet M, Touati G, Grabowski P, Abitbol M (2003). Splicing error in E1alpha pyruvate dehydrogenase mRNA caused by novel intronic mutation responsible for lactic acidosis and mental retardation. J Biol Chem.

[pbio-0030158-b51] Chen CD, Kobayashi R, Helfman DM (1999). Binding of hnRNP H to an exonic splicing silencer is involved in the regulation of alternative splicing of the rat beta-tropomyosin gene. Genes Dev.

[pbio-0030158-b52] Modafferi EF, Black DL (1997). A complex intronic splicing enhancer from the c-src pre-mRNA activates inclusion of a heterologous exon. Mol Cell Biol.

[pbio-0030158-b53] Chou MY, Rooke N, Turck CW, Black DL (1999). hnRNP H is a component of a splicing enhancer complex that activates a c-src alternative exon in neuronal cells. Mol Cell Biol.

[pbio-0030158-b54] Min H, Chan RC, Black DL (1995). The generally expressed hnRNP F is involved in a neural-specific pre-mRNA splicing event. Genes Dev.

[pbio-0030158-b55] Buratti E, Baralle M, De Conti L, Baralle D, Romano M (2004). hnRNP H binding at the 5′ splice site correlates with the pathological effect of two intronic mutations in the NF-1 and TSHbeta genes. Nucleic Acids Res.

[pbio-0030158-b56] Cartegni L, Wang J, Zhu Z, Zhang MQ, Krainer AR (2003). ESEfinder: A web resource to identify exonic splicing enhancers. Nucleic Acids Res.

[pbio-0030158-b57] Fu XD, Mayeda A, Maniatis T, Krainer AR (1992). General splicing factors SF2 and SC35 have equivalent activities in vitro, and both affect alternative 5′ and 3′ splice site selection. Proc Natl Acad Sci U S A.

[pbio-0030158-b58] Mayeda A, Krainer AR (1992). Regulation of alternative pre-mRNA splicing by hnRNP A1 and splicing factor SF2. Cell.

[pbio-0030158-b59] Mayeda A, Helfman DM, Krainer AR (1993). Modulation of exon skipping and inclusion by heterogeneous nuclear ribonucleoprotein A1 and pre-mRNA splicing factor SF2/ASF. Mol Cell Biol.

[pbio-0030158-b60] Caceres JF, Stamm S, Helfman DM, Krainer AR (1994). Regulation of alternative splicing in vivo by overexpression of antagonistic splicing factors. Science.

[pbio-0030158-b61] Yang X, Bani MR, Lu SJ, Rowan S, Ben-David Y (1994). The A1 and A1B proteins of heterogeneous nuclear ribonucleoparticles modulate 5′ splice site selection in vivo. Proc Natl Acad Sci U S A.

[pbio-0030158-b62] Damgaard CK, Tange TO, Kjems J (2002). hnRNP A1 controls HIV-1 mRNA splicing through cooperative binding to intron and exon splicing silencers in the context of a conserved secondary structure. RNA.

[pbio-0030158-b63] Eperon IC, Makarova OV, Mayeda A, Munroe SH, Caceres JF (2000). Selection of alternative 5′ splice sites: Role of U1 snRNP and models for the antagonistic effects of SF2/ASF and hnRNP A1. Mol Cell Biol.

[pbio-0030158-b64] Marchand V, Mereau A, Jacquenet S, Thomas D, Mougin A (2002). A Janus splicing regulatory element modulates HIV-1 tat and rev mRNA production by coordination of hnRNP A1 cooperative binding. J Mol Biol.

[pbio-0030158-b65] Zhu J, Mayeda A, Krainer AR (2001). Exon identity established through differential antagonism between exonic splicing silencer-bound hnRNP A1 and enhancer-bound SR proteins. Mol Cell.

[pbio-0030158-b66] Su AI, Cooke MP, Ching KA, Hakak Y, Walker JR (2002). Large-scale analysis of the human and mouse transcriptomes. Proc Natl Acad Sci U S A.

[pbio-0030158-b67] Su AI, Wiltshire T, Batalov S, Lapp H, Ching KA (2004). A gene atlas of the mouse and human protein-encoding transcriptomes. Proc Natl Acad Sci U S A.

[pbio-0030158-b68] Wollerton MC, Gooding C, Wagner EJ, Garcia-Blanco MA, Smith CW (2004). Autoregulation of polypyrimidine tract binding protein by alternative splicing leading to nonsense-mediated decay. Mol Cell.

[pbio-0030158-b69] Blanchette M, Chabot B (1999). Modulation of exon skipping by high-affinity hnRNP A1-binding sites and by intron elements that repress splice site utilization. EMBO J.

[pbio-0030158-b70] Sureau A, Gattoni R, Dooghe Y, Stevenin J, Soret J (2001). SC35 autoregulates its expression by promoting splicing events that destabilize its mRNAs. EMBO J.

[pbio-0030158-b71] Okazaki Y, Furuno M, Kasukawa T, Adachi J, Bono H (2002). Analysis of the mouse transcriptome based on functional annotation of 60,770 full-length cDNAs. Nature.

[pbio-0030158-b72] Xu Q, Modrek B, Lee C (2002). Genome-wide detection of tissue-specific alternative splicing in the human transcriptome. Nucleic Acids Res.

[pbio-0030158-b73] Grabowski PJ (1998). Splicing regulation in neurons: Tinkering with cell-specific control. Cell.

[pbio-0030158-b74] Black DL (2000). Protein diversity from alternative splicing: A challenge for bioinformatics and post-genome biology. Cell.

[pbio-0030158-b75] Zhang L, Ashiya M, Sherman TG, Grabowski PJ (1996). Essential nucleotides direct neuron-specific splicing of gamma 2 pre-mRNA. RNA.

[pbio-0030158-b76] Nasim FH, Spears PA, Hoffmann HM, Kuo HC, Grabowski PJ (1990). A Sequential splicing mechanism promotes selection of an optimal exon by repositioning a downstream 5′ splice site in preprotachykinin pre-mRNA. Genes Dev.

[pbio-0030158-b77] Arhin GK, Boots M, Bagga PS, Milcarek C, Wilusz J (2002). Downstream sequence elements with different affinities for the hnRNP H/H′ protein influence the processing efficiency of mammalian polyadenylation signals. Nucleic Acids Res.

[pbio-0030158-b78] Bagga PS, Arhin GK, Wilusz J (1998). DSEF-1 is a member of the hnRNP H family of RNA-binding proteins and stimulates pre-mRNA cleavage and polyadenylation in vitro. Nucleic Acids Res.

[pbio-0030158-b79] Siomi H, Dreyfuss G (1995). A nuclear localization domain in the hnRNP A1 protein. J Cell Biol.

[pbio-0030158-b80] Moore MJ, Query CC (2000). Joining of RNAs by splinted ligation. Methods Enzymol.

[pbio-0030158-b81] Ashiya M, Grabowski PJ (1997). A neuron-specific splicing switch mediated by an array of pre-mRNA repressor sites: Evidence of a regulatory role for the polypyrimidine tract binding protein and a brain-specific PTB counterpart. RNA.

[pbio-0030158-b82] Veraldi KL, Arhin GK, Martincic K, Chung-Ganster LH, Wilusz J (2001). hnRNP F influences binding of a 64-kilodalton subunit of cleavage stimulation factor to mRNA precursors in mouse B cells. Mol Cell Biol.

